# Interleukin-33 Signaling Controls the Development of Iron-Recycling Macrophages

**DOI:** 10.1016/j.immuni.2020.03.006

**Published:** 2020-05-19

**Authors:** Yuning Lu, Gemma Basatemur, Ian C. Scott, Davide Chiarugi, Marc Clement, James Harrison, Ravin Jugdaohsingh, Xian Yu, Stephen A. Newland, Helen E. Jolin, Xuan Li, Xiao Chen, Monika Szymanska, Guttorm Haraldsen, Gaby Palmer, Padraic G. Fallon, E. Suzanne Cohen, Andrew N.J. McKenzie, Ziad Mallat

**Affiliations:** 1 Division of Cardiovascular Medicine, University of Cambridge, Cambridge, UK; 2 Translational Sciences and Experimental Medicine, Early Respiratory and Immunology, Biopharmaceuticals R&D, AstraZeneca, Cambridge, UK; 3 The Wellcome-MRC Institute of Metabolic Science-Metabolic Research Laboratories, Cambridge, UK; 4 Department of Veterinary Medicine, University of Cambridge, Cambridge, UK; 5 Medical Research Council Laboratory of Molecular Biology, Cambridge, UK; 6 Department of Cardiology, Union Hospital, Tongji Medical College, Huazhong University of Science and Technology, China; 7 Department of Pathology, Oslo University Hospital Rikshospitalet, Norway; 8 Division of Rheumatology, Department of Internal Medicine Specialties, University Hospitals of Geneva, Geneva, Switzerland; 9 Department of Pathology-Immunology, University of Geneva School of Medicine, Geneva, Switzerland; 10 School of Medicine, Trinity Biomedical Sciences Institute, Trinity College Dublin, Dublin 2, Ireland; 11 Bioscience Asthma, Early Respiratory and Immunology, BioPharmaceuticals R&D, AstraZeneca, Cambridge, UK; 12 Institut National de la Santé et de la Recherche Médicale, Paris Cardiovascular Research Center, Paris, France

**Keywords:** interleukins, red pulp macrophage, iron metabolism, erythrophagocytosis, interleukin-33, interleukin-33 receptor

## Abstract

Splenic red pulp macrophages (RPMs) contribute to erythrocyte homeostasis and are required for iron recycling. Heme induces the expression of SPIC transcription factor in monocyte-derived macrophages and promotes their differentiation into RPM precursors, pre-RPMs. However, the requirements for differentiation into mature RPMs remain unknown. Here, we have demonstrated that interleukin (IL)-33 associated with erythrocytes and co-cooperated with heme to promote the generation of mature RPMs through activation of the MyD88 adaptor protein and ERK1/2 kinases downstream of the IL-33 receptor, IL1RL1. IL-33- and IL1RL1-deficient mice showed defective iron recycling and increased splenic iron deposition. Gene expression and chromatin accessibility studies revealed a role for GATA transcription factors downstream of IL-33 signaling during the development of pre-RPMs that retained full potential to differentiate into RPMs. Thus, IL-33 instructs the development of RPMs as a response to physiological erythrocyte damage with important implications to iron recycling and iron homeostasis.

## Highlights

• IL-33 signaling promotes development of monocyte-derived red pulp macrophages (RPMs)
• *Il33*^*−/−*^ and *Il1rl1*^*−/−*^ mice have decreased RPMs and splenic erythrophagocytosis
• ERK activation is required for RPM development and is potentiated by hemin and IL-33
• GATA2 instructs RPM development and is aberrant in *Il1rl1*^*−/−*^ RPM precursors

## Graphical Abstract

**Figure:**
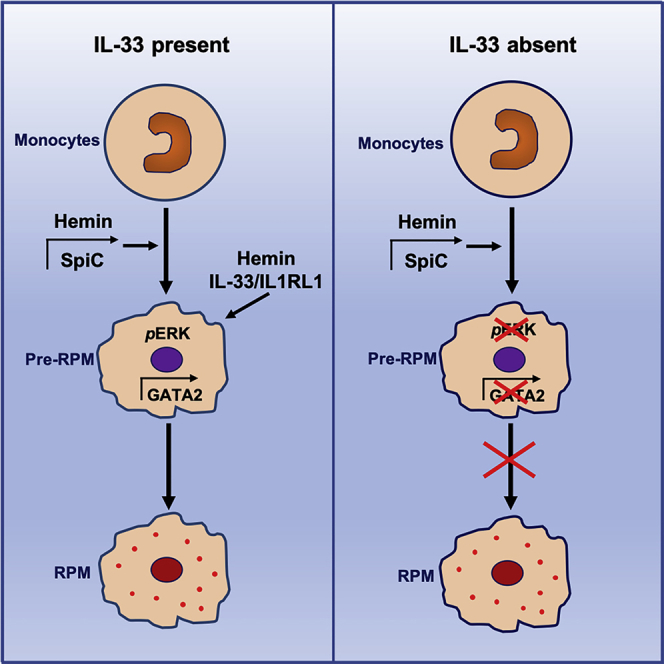

## Teaser

Splenic red pulp macrophages (RPMs) are critical for iron recycling. Here, Lu et al. implicate a role for IL-33 signaling in the regulation of GATA2, which controls the transition of monocytes to produce pre-RPMs that are competent to terminally differentiate into mature RPMs.

## Introduction

The development of tissue-resident macrophages from yolk-sac-derived pre-macrophages (Mass et al., 2016) or bone marrow-derived monocytes (Scott et al., 2016) requires local specification by instructive signals coming from the tissue microenvironment (Amit et al., 2016, Gautier et al., 2014, Okabe and Medzhitov, 2014). This is the case for some locally generated metabolites like retinoic acid, which is required for the polarization of peritoneal macrophages, and heme, which plays an essential role in the commitment of monocytes to a pre-red pulp macrophage (RPM) phenotype, dependent on the transcription factor SPIC (Amit et al., 2016, Haldar et al., 2014, Okabe and Medzhitov, 2014). An important physiological function of RPMs is the regulation of iron metabolism through erythrocyte clearance and iron recycling. RPMs phagocytose other blood-borne particulates and express several innate immune receptors, and may therefore be involved in the regulation of immune-inflammatory responses. RPMs are also involved in type I interferon production in response to parasites (*P. Chabaudi*) and are able to cross-prime early effector T cell responses against viruses, and may therefore play a role in the defense against infections (Borges da Silva et al., 2015, Enders et al., 2020, Kurotaki et al., 2015).

Cytokines are important modulators of macrophage phenotype and function during inflammation and tissue repair (Bosurgi et al., 2017, Howangyin et al., 2016, Ip et al., 2017, Jenkins et al., 2011, Minutti et al., 2017). However, the requirement for a local source of cytokines during the homeostatic development and local specification of tissue-resident macrophages is not established (Amit et al., 2016). For example, local production of interleukin (IL)-34 in brain and skin is required for the development of microglia and Langerhans cells (Wang et al., 2012). However, in this case, IL-34 mostly acts as a local colony stimulating factor-1 receptor (CSF1R) ligand required for monocyte and/or macrophage survival and proliferation, rather than being involved in the functional specialization to the microglial or Langerhans cell phenotype. Transforming growth factor-β1 (TGF-β1) is also essential for the acquisition of microglia and Langerhans cell signatures (Butovsky et al., 2014). However, the source of TGF-β1 has not been defined, and it is still unknown whether TGF-β1 signaling is required for microglia development in a cell-autonomous manner. Furthermore, granulocyte macrophage-colony stimulating factor (GM-CSF) plays a specific role in the development of alveolar macrophages (Guilliams et al., 2013, Hashimoto et al., 2013), though it is unclear whether a specific local source of GM-CSF is required for this effect. In addition, IL-10 is essential for the control of macrophage inflammatory responses through metabolic reprogramming of macrophages, inhibition of mammalian target of rapamycin (mTOR) signaling, and promotion of mitophagy (Ip et al., 2017). IL-4 is also involved in the proliferation of tissue-resident macrophages in response to type 2 inflammation (Jenkins et al., 2011) and is required together with IL-13 for the induction of a tissue repair macrophage phenotype after type 2-mediated macrophage activation (Bosurgi et al., 2017, Minutti et al., 2017) and aseptic ischemic injury (Howangyin et al., 2016). However, those cytokines are not required for the establishment of tissue-resident macrophages under homeostatic conditions.

IL-33 is a member of the IL-1 cytokine family, which plays a crucial role in initiation and amplification of immune responses to combat injury and infection (Cayrol and Girard, 2018, Liew et al., 2016, Scott et al., 2018b). IL-33 is constitutively expressed in the nucleus of epithelial cells in barrier tissues and endothelial cells of blood vessels. On release from damaged cells, IL-33 activates many different immune cell types via its receptor, interleukin receptor-like 1 (IL1RL1, also known as ST2). However, direct roles for IL-33 activity in regulating cellular differentiation and functions of mononuclear cell functions have remained poorly understood. It has been reported that IL1RL1 is not expressed on macrophages at steady state but can be induced (Kearley et al., 2015), and IL-33 signaling has been implicated in regulating osteoclast and macrophage foam cell formation (Kiyomiya et al., 2015, McLaren et al., 2010, Velickovic et al., 2015, Zaiss et al., 2011). Recent studies have highlighted the role of stromal-derived IL-33 in the generation of a pro-tumorigenic M2-like macrophage phenotype (Andersson et al., 2018) and the role of astrocyte-derived IL-33 in promoting microglial-dependent synapse engulfment and depletion, thereby affecting neural circuit function (Vainchtein et al., 2018).

The identity of the instructive signals required for the differentiation of pre-RPMs into a mature RPM phenotype remains unknown. Given the prominent roles of cytokines in the modulation of macrophage phenotype and function, we hypothesized their potential involvement in the generation of RPMs. Here, we show that IL-33 signaling plays a previously unappreciated role in the development of iron-recycling macrophages.

## Results

**Figure 1:**
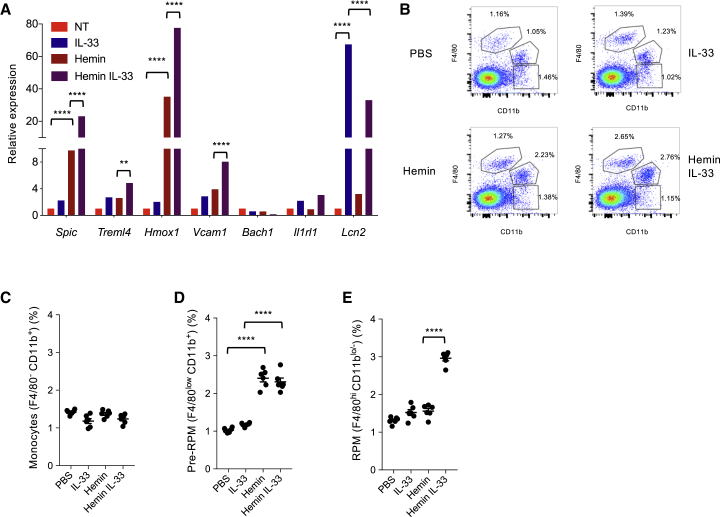
IL-33 Upregulates Hemin-Induced *Spic* Expression and Promotes the Development of a Red Pulp Macrophage (RPM) Phenotype *In Vitro* and *In Vivo* (A) Gene expression in mouse bone-marrow-derived macrophages stimulated *in vitro* for 4 days with hemin (40 μM), IL-33 (10 ng/mL), or a combination of hemin + IL-33, compared to no treatment (NT). Data represent mean ± SEM and are representative of five independent experiments. ^∗∗^p < 0.01, ^∗∗∗∗^p < 0.001. (B–E) Representative examples (B) and quantification (among CD45^+^ CD11c^low^ Ly6G^low^ NK1.1^low^ SSC-A^low^ cells) of flow cytometry staining for splenic monocytes (CD11b^+^ F4/80^−^) (C), pre-RPMs (CD11b^+^ F4/80^lo^) (D), and RPMs (CD11b^lo/−^ F4/80^hi^) (E) in mice injected intraperitoneally once a day for 3 days with either phosphate-buffered saline (PBS), IL-33 (1 μg), hemin (500 μg), or IL-33 + hemin. ^∗∗∗∗^p < 0.001. Data representative of at least five independent experiments. Please also see Figure S1.

We used previously validated assays of hemin-mediated induction of monocyte differentiation into an iron-recycling macrophage phenotype (Haldar et al., 2014) to survey potential roles of type 1 and type 2 cytokines on this process in the presence and absence of heme. We found that only IL-33 produced a significant increase in gene expression of markers of RPM phenotype (Figures 1A and S1A). IL-33 alone had a limited impact. However, co-stimulation of macrophages with IL-33 and hemin led to a substantial increase of hemin-induced expression of *Spic* (Figure 1A). Other prototypic genes of iron-recycling macrophages including *Treml4*, *Vcam1*, and *Hmox1* were significantly upregulated (Figure 1A), whereas the expression of *Bach1*, a transcriptional repressor of *Spic* (Haldar et al., 2014), was not altered (Figure 1A). Of note, IL-33 alone substantially induced the expression of *Lcn2* (Figure 1A), which encodes a protein (Lipocalin-2) that regulates iron transport and limits microbial growth through sequestration of iron-containing siderophores (Xiao et al., 2017), further supporting a role for IL-33 in the induction of an iron-recycling RPM phenotype. We obtained similar results with IL-33 in human CD14^+^ monocyte-derived macrophages (Figure S1B).

We then purified splenic monocytes, pre-RPMs, and RPMs, and stimulated them *in vitro* with hemin in the presence or absence of IL-33. First, we confirmed using the *Spic*^*igfp/igfp*^ reporter mice (Haldar et al., 2014) that splenic RPMs (CD11b^lo/−^ F4/80^hi^) are *Spic*-EGFP^hi^, whereas splenic pre-RPMs (CD11b^hi^ F4/80^lo^) are *Spic*-EGFP^lo/int^ (Figure S1C) and splenic monocytes (CD11b^hi^ F4/80^−^) express little SPIC (not shown). This was further confirmed by gene expression analysis on sorted populations of splenic monocytes, pre-RPMs, and RPMs, which revealed intermediate expression of RPM-associated genes in pre-RPMs compared to monocytes and RPMs (Figure S1D). This supports the previous demonstration of a developmental continuum between these three cell subsets (Haldar et al., 2014). Furthermore, we found that IL-33 had a substantial impact on hemin-induced *Spic* expression only in purified pre-RPMs (Figure S1E), with no detectable effect in splenic monocytes (Figure S1E) and splenic RPMs (data not shown). These results suggested a potential requirement of IL-33 to induce the differentiation of pre-RPMs into RPMs. To test this hypothesis *in vivo*, we treated mice with hemin, IL-33, or both, and assessed the induction of pre-RPMs and RPMs. In agreement with previous work (Haldar et al., 2014), we found that hemin administration for 3 days increased the population of CD11b^hi^ *Spic*-EGFP^lo^ F4/80^lo^ (Figures 1B–1E and S1F–S1H) pre-RPMs but was unable to induce the generation of CD11b ^lo/−^ F4/80^hi^ *Spic*-EGFP^hi^ RPMs (Figures 1B–1E and S1F–S1H). In contrast, co-administration of IL-33 with hemin led to a substantial increase of the CD11b ^lo/−^ *Spic*-EGFP^hi^ F4/80^hi^ RPMs (Figures 1B–1E and S1F–S1H).

**Figure 2:**
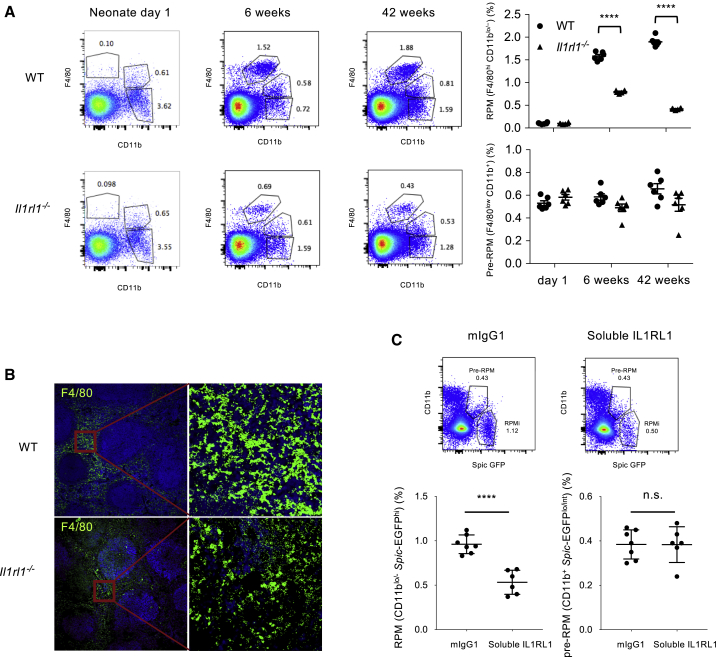
IL1RL1 Signaling Controls the Development of Splenic Red Pulp Macrophages (RPMs) (A) Quantification (percentage among CD11c^low^ Ly6G^low^ NK1.1^low^ SSC-A^low^ cells) of splenic pre-RPMs (CD11b^hi^ F4/80^lo^) and RPMs (CD11b^lo/−^ F4/80^hi^) in neonates (Day 1), young (6 weeks), and old (42 weeks) WT and *Il1rl1*^*−/−*^ mice. Each dot represents a separate mouse. ^∗∗∗∗^p < 0.001. (B) Staining of RPMs (F4/80 shown in green) in spleen sections of 42-week WT and *Il1rl1*^*−/−*^ mice. (C) Flow cytometry staining and quantification of splenic RPMs (CD11b^lo/^*Spic*-EGFP^hi^) and pre-RPMs (CD11b^+^*Spic*-EGFP^lo/int^) (percentages among CD11c^low^ Ly6G^low^ NK1.1^low^ SSC-A^low^ cells) after 6 weeks of treatment of *Spic*^*igfp/igfp*^ reporter mice with control murine IgG1 (mIgG1) or soluble IL1RL1-murine Fc fusion protein (soluble IL1RL1). Each dot in the quantification panels represents a separate mouse. Data represent mean ± SEM. ^∗∗∗∗^p < 0.001. Please also see Figures S2 and S3.

The IL-33 receptor, IL1RL1, mediates IL-33 signaling. IL1RL1 deficiency did not alter hemin-induced gene expression in monocyte-derived macrophages *in vitro*, but completely abrogated IL-33-dependent upregulation of *Spic* and other RPM-associated genes (Figure S2A). To address the requirement of IL1RL1 in RPM development *in vivo*, we analyzed IL1RL1-deficient mice (Figure S2B for IL1RL1 expression on RPMs) at different time points. We found that both the percentage of RPMs (Figure 2A) and SPIC expression (Figures S2C and S2D) increased with age from neonatal days 1 and 2 to 4 weeks or 6 weeks of age in wild-type (WT) mice. IL1RL1 deficiency had no impact on RPM number in neonates but resulted in a significant ∼50% and ∼80% reduction in RPM number compared to WT mice at 6 and 42 weeks of age, respectively (Figures 2A and S2E). This was supported by a substantial reduction of F4/80^+^ staining in spleen sections of IL1RL1-deficient mice compared to WT mice at 42 weeks (Figure 2B). There was no impact of IL1RL1 deficiency on other splenic cell types (Figure S2F) or liver Kupffer macrophages (data not shown). Consistent with these data, we found that treatment of adult *Spic*^*igfp/igfp*^ reporter mice with murine soluble IL1RL1-Fc fusion protein for 6 weeks, to block IL-33 signaling, significantly reduced the percentage of *Spic*-EGFP^hi^ CD11b^lo/−^ RPMs (without affecting pre-RPM number) in comparison with mice that received murine IgG1 (Figures 2C). We then generated mixed bone marrow chimeras (50% CD45.2 WT or 50% IL1RL1-deficient CD45.2 bone marrow + 50% CD45.1 WT bone marrow into irradiated CD45.2 WT mice) to address the cell-autonomous requirement of IL1RL1 expression in bone-marrow-derived monocytes for their differentiation into RPMs (Figure S3A). We found that splenic monocytes and pre-RPMs were generated at similar frequencies from the CD45.1 and CD45.2 bone marrow, under the basal state or after combined hemin and IL-33 stimulation, whatever the status of IL1RL1 expression (Figure S3B). By contrast, most RPMs in the 50% CD45.2 IL1RL1-deficient + 50% CD45.1 WT group originated from the CD45.1 bone marrow (Figure S3C), indicating a profound alteration of RPM generation in the absence of IL1RL1 expression in monocytes and pre-RPMs. We also lethally irradiated CD45.1 WT mice and reconstituted them with CD45.2 bone marrow originating from either WT or IL1RL1-deficient mice. Similarly, we found a marked defect in RPM generation from the CD45.2 IL1RL1-deficient bone marrow compared to CD45.2 WT bone marrow (Figures S3D–S3F).

**Figure 3:**
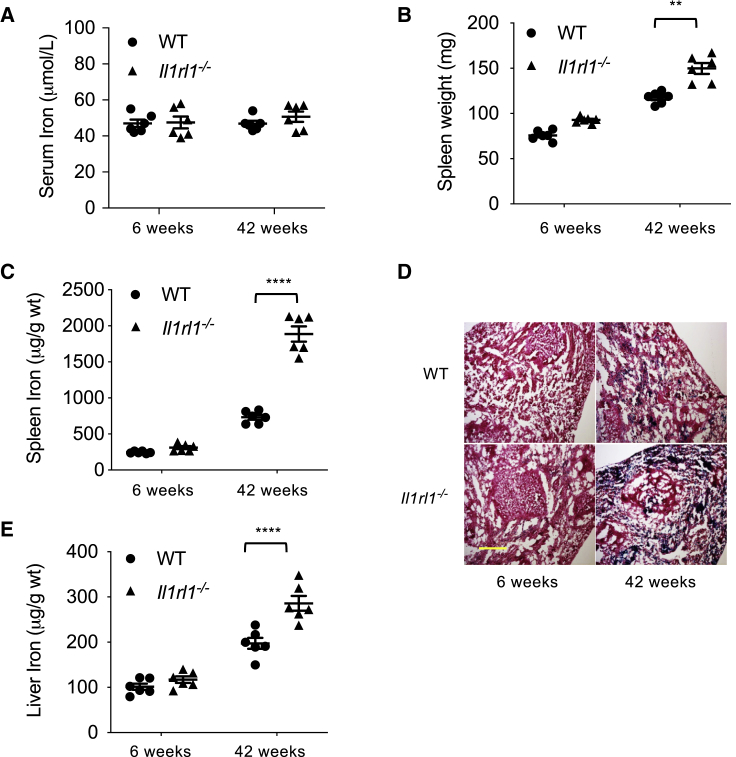
Impact of IL1RL1 Signaling on the Iron-Recycling Capacity of Splenic Red Pulp Macrophages (RPMs) (A–C) Quantification of serum iron (A), spleen weight (B), and spleen iron (C) in young (6 weeks) and old (42 weeks) WT and *Il1rl1*^*−/−*^ mice. (D) Representative examples (6 mice per group) of Perl’s blue staining (to detect iron) in spleen sections of young (6 weeks) and old (42 weeks) WT and *Il1rl1*^*−/−*^ mice. Scale unit: 300μm. (E) Quantification of liver iron in young (6 weeks) and old (42 weeks) WT and *Il1rl1*^*−/−*^ mice. Each dot represents a separate mouse. ^∗∗^p < 0.01, ^∗∗∗∗^p < 0.001. Please also see Figure S4.

We then addressed the functional impact of these findings. We found that the uptake of PKH26-labeled red blood cells (RBCs) *in vivo* was reduced in RPMs, but not pre-RPMs or other cell types, in the absence of IL1RL1 (Figures S4A and S4B), suggesting defective iron-recycling capacity. Next, we measured iron stores in young (6 weeks old) and old (42 weeks old) WT and IL1RL1-deficient mice. While we found no difference in serum iron concentrations between mouse groups (Figure 3A), we detected a significant increase of spleen weight (Figure 3B) and a 3-fold increase of splenic tissue iron concentration in 42-week-old IL1RL1-deficient mice compared to WT controls (Figure 3C). Substantial iron accumulation was confirmed histologically on splenic tissue sections (Figure 3D). In case of defective iron recycling in spleen, the liver can increase its iron-recycling capacity through increased recruitment of circulating monocytes and their differentiation into iron-recycling macrophages (Theurl et al., 2016). We found iron concentrations in liver were also substantially increased in old IL1RL1-deficient compared to WT controls (Figure 3E), suggesting that the iron-recycling capacity of liver macrophages was overwhelmed. Old IL1RL1-deficient mice (42 weeks) did not show evidence of anemia (Figure S4C). However, serum ferritin concentration was substantially higher in old IL1RL1-deficient mice compared to WT controls (Figure S4C). Taken together, our data identify a major role for the IL1RL1 signaling pathway in the differentiation of monocytes to iron-recycling RPMs.

**Figure 4:**
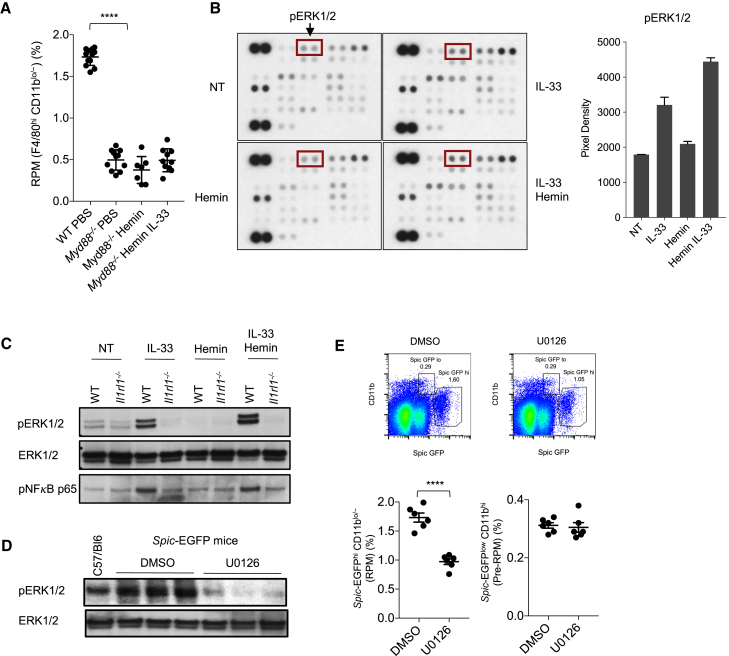
MyD88 and ERK1/2 Signaling Downstream of IL-33 Control the Development of Red Pulp Macrophages (RPMs) (A) Quantification of RPMs (percentage among CD11c^low^ Ly6G^low^ NK1.1^low^ SSC-A^low^ cells) in spleens of WT and *Myd88*^*−/−*^ mice with or without treatment with hemin alone or IL-33 + hemin (one daily injection for 3 days; see STAR Methods). (B) Human macrophages (generated by culture of CD14^+^ monocytes from blood) treated for 5 min with phosphate-buffered saline (NT), hemin (40 μM), IL-33 (10 ng/mL), or a combination of hemin + IL-33, and assessed using a phospho-kinase array. pERK1/2 is shown in red boxes. Quantification of the intensity of ERK1/2 phosphorylation signal (pixel density) was performed using ImageJ. Data represent mean ± SEM and are representative of three independent experiments. (C) Mouse bone-marrow-derived macrophages of WT and *Il1rl1*^*−/−*^ mice treated *in vitro* for 5 min with PBS (NT), hemin, IL-33, or a combination of hemin + IL-33, and assessed for pERK1/2, ERK1/2, and phospho-p65 (NF-κB) using western blotting. Semi-quantification of signal intensity (mean ± SEM) of ERK1/2 phosphorylation (pERK/Total ERK) using ImageJ yielded WT IL-33: 0.74 ± 0.02 versus WT IL-33+Hemin: 1.24 ± 0.09, p < 0.05. Semi-quantification of signal intensity (mean ± SEM) of p65 phosphorylation using ImageJ yielded WT IL-33: 11750 ± 3500 versus WT IL-33+Hemin: 12607 ± 4500, p = 0.09. (D and E) C57/Bl6 *Spic*^*igfp/igfp*^ reporter mice were treated with ERK1/2 inhibitor U0126 or DMSO control for 6 weeks (see STAR Methods). ERK1/2 phosphorylation in splenic extracts (D), and splenic CD11b^low^*Spic*-EGFP^hi^ RPMs and CD11b^+^*Spic*-EGFP^int^ pre-RPMs (percentages among CD11c^low^ Ly6G^low^ NK1.1^low^ SSC-A^low^ cells) (E), were quantified at the end of the experiment. A control non-reporter untreated mouse (C57/Bl6) is included as control in (D). Each lane in (C) and (D) represents a separate mouse. Each dot in (E) represents a separate mouse. ^∗∗∗∗^p < 0.001.

We sought to examine which intracellular signaling pathways are involved in the process downstream of IL1RL1 activation. *Myd88*^*−/−*^ mice displayed reduced numbers of RPMs (but not monocytes or pre-RPMs; data not shown) at steady state and did not increase their RPMs after *in vivo* administration of IL-33 and hemin (Figure 4A). Several signaling pathways are activated downstream of MyD88 following IL1RL1 engagement by IL-33 (Liew et al., 2016, Pinto et al., 2015). Using a human phosphokinase assay, we found that the combination of hemin and IL-33 led to increased ERK1/2 phosphorylation in monocyte-derived macrophages *in vitro*, compared to IL-33 alone (Figure 4B). MSK1/2 phosphorylation was also marginally increased in presence of IL-33 and hemin; however, there was no combined effect on any other signaling pathways surveyed (data not shown). We confirmed the effect of IL-33 and hemin on ERK1/2 phosphorylation in mouse bone marrow monocyte-derived macrophages (Figure 4C). Next, we investigated the impact of *in vivo* inhibition of ERK1/2 for 6 weeks on RPM number. The MEK inhibitor U0126 prevented ERK1/2 phosphorylation in spleen lysates (Figure 4D) and reduced RPM (CD11b ^lo/−^ F4/80^hi^ *Spic*-EGFP^hi^) number compared to vehicle-treated mice (Figure 4E), without altering pre-RPMs (CD11b^hi^ *Spic*-EGFP^lo^ F4/80^lo^; Figure 4E).

**Figure 5:**
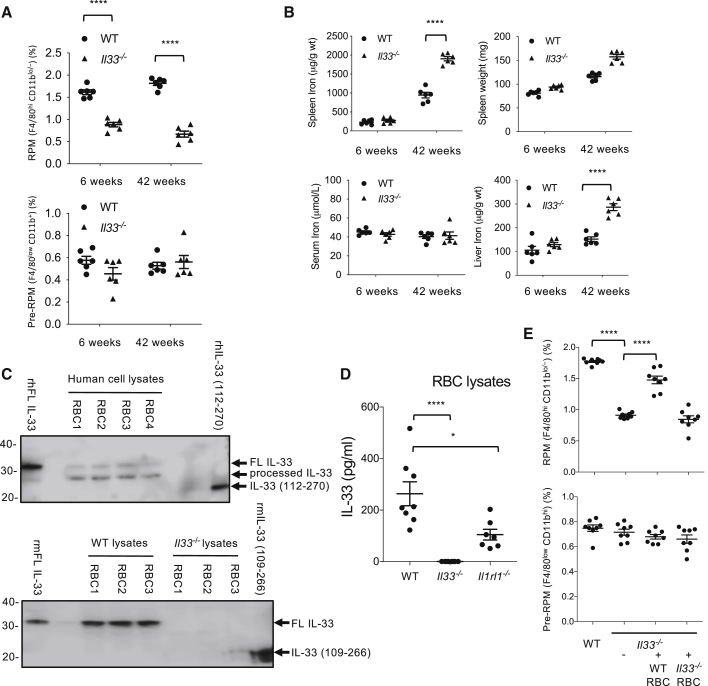
Red-Blood-Cell-Derived IL-33 Controls the Development of Splenic Red Pulp Macrophages (RPMs) (A) Quantification of RPMs and pre-RPMs (percentage among CD11c^low^ Ly6G^low^ NK1.1^low^ SSC-A^low^ cells) in spleens of young (6 weeks) and old (42 weeks) WT and *Il33*^*−/−*^ mice. (B) Quantification of serum iron, spleen weight, spleen iron, and liver iron in young (6 weeks) and old (42 weeks) WT and *Il33*^*−/−*^ mice. (C) Detection of IL-33 in human (top panel) and mouse (lower panel) red blood cell (RBC) lysates using western blotting. Recombinant full-length and processed human and mouse IL-33 are included as positive controls. RBC lysates from *Il33*^*−/−*^ mice are included as negative controls. Each lane is from a separate mouse or individual, representative of at least five independent experiments. (D) Quantification of IL-33 protein in RBC lysates of WT, *Il33*^*−/−*^ (negative control), and *Il1rl1*^*−/−*^ mice. (E) Quantification (percentage among CD11c^low^ Ly6G^low^ NK1.1^low^ SSC-A^low^ cells) of splenic pre-RPMs (CD11b^hi^ F4/80^lo^) and RPMs (CD11b^lo/−^ F4/80^hi^) by flow cytometry in WT and *Il3*^*−/−*^ mice. Some *Il33*^*−/−*^ mice were reconstituted with either WT or *Il33*^*−/−*^ RBCs (see STAR Methods) prior to assessment of splenic pre-RPMs and RPMs. Each dot in (A), (B), (D), and (E) represents a separate mouse. ^∗∗∗∗^p < 0.001. Please also see Figure S5 and S6.

We next addressed the role of endogenous IL-33 in the modulation of RPMs. Similar to IL1RL1-deficient mice, young and old IL-33-deficient mice displayed a substantial reduction of RPMs compared to WT controls (Figures 5A, S2E, and S5A), and we were able to partially restore RPM counts in IL-33-deficient mice after treatment for 4 days with IL-33 alone (Figure S5B), an effect that was further enhanced by the addition of hemin (Figure S5B). Like IL1RL1-deficient mice, old IL-33-deficient mice showed a significant increase of spleen weight, splenic tissue iron concentration, and liver iron accumulation compared to control mice (Figure 5B), strongly suggesting reduced iron-recycling capacity in the absence of IL-33. IL-33-deficient rats also showed a substantial reduction in CD11b^lo^EMR1^hi^ splenic macrophages (EMR1 is the homolog of F4/80) compared to control animals (Figure S5C), further supporting a role for IL-33 signaling in the development and/or maintenance of RPMs.

Next, we investigated the potential source of endogenous IL-33 that could modulate RPMs. As IL-33 and hemin co-operated to promote the iron-recycling macrophage phenotype, we hypothesized that IL-33 might be co-expressed with hemin in erythrocytes. We therefore analyzed IL-33 expression in RBCs and their progenitors. We detected a ∼31 kDa full-length form of IL-33 protein (Cayrol et al., 2018, Scott et al., 2018a) in WT, but not IL-33-deficient murine erythrocyte lysates (Figure 5C). Human IL-33 was weakly detectable in full-length form but was predominantly detected as a ∼29 kDa form in human erythrocyte lysates (Figure 5C; and data not shown). This ∼29 kDa form was also seen in the recombinant full-length control (Figures 5C and S6A) and was detected in highly enriched CD235a^+^ human erythrocytes using two additional anti-IL-33 antibodies (Figure S6A). IL-33 protein expression in human erythrocyte lysates was confirmed by ELISA (Figures 5D and S6B). We explored mechanisms for the association of IL-33 by erythrocytes. One possibility is that erythroid precursors express IL-33. Another possibility is that erythrocytes bind IL-33, either via IL1RL1-dependent or -independent pathways. Using *Il33*-Citrine reporter mice, we found that only ∼5% of erythrocyte progenitors expressed IL-33 (Figure S6C), broadly consistent with low amounts of IL-33 in mature erythrocytes. Among bone marrow cells, IL1RL1 was preferentially expressed in erythroid progenitors (stage B erythroblasts; Koulnis et al., 2011), but not mature erythrocytes (Figure S6D; and data not shown), and was capable of binding and internalizing recombinant IL-33 (Figure S6E). Consistent with this observation, we found that IL-33 was significantly lower in erythrocyte lysates of IL1RL1-deficient mice compared to WT mice (Figure 5D). We then addressed the *in vivo* relevance of erythrocyte-associated IL-33 to the development of RPMs. Notably, reconstitution of IL-33-deficient mice with WT, but not IL-33-deficient erythrocytes, predominantly restored RPMs without affecting pre-RPM numbers (Figure 5E), whereas reconstitution of IL-33-deficient mice with erythrocytes from IL1RL1-deficient mice partially restored RPM numbers (Figure S6F). Thus, erythrocytes can provide both heme and IL-33 to induce the development of monocyte-derived RPMs. However, it is plausible that IL-33 expressed by other cell types in the spleen may also contribute IL-33 to enable the development of RPMs.

**Figure 6:**
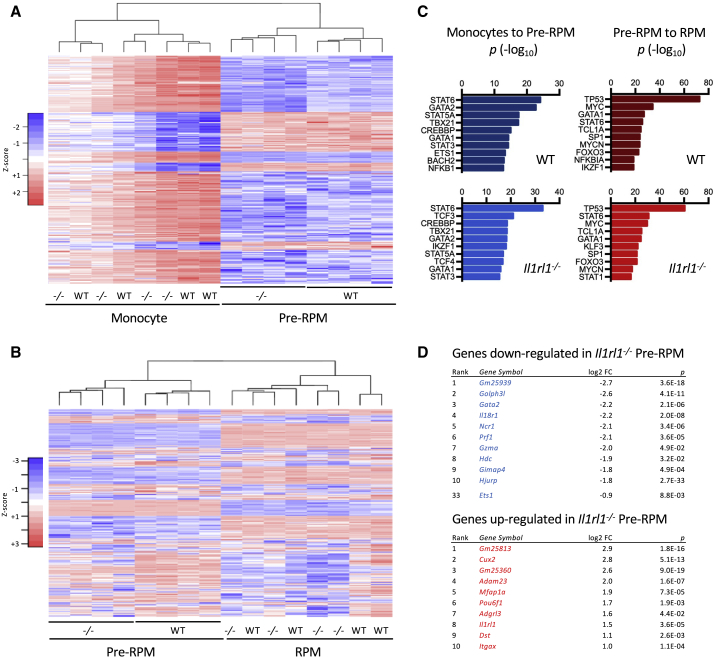
GATA2 Is Implicated in the Development of Splenic Red Pulp Macrophages (RPMs) and Differentially Expressed in WT and *Ilrl1*^*−/−*^ Pre-RPMs (A)) Heatmap showing relative expression of genes differentially expressed between WT monocytes and WT pre-RPMs (693 genes, log2 fold change > 1, adjusted p value < 0.01) in splenic monocytes (CD11b^hi^ F4/80^−^) and pre-RPMs (CD11b^hi^ F4/80^lo^) from WT and *Il1rl1*^*−/−*^ mice; n = 4 separate mice per group. (B) Heatmap showing relative expression of genes differentially expressed between WT pre-RPMs and WT RPMs (3079 genes, log2 fold change > 1, adjusted p value < 0.01) in pre-RPMs (CD11b^hi^ F4/80^lo^) and RPMs (CD11b^lo/−^ F4/80^hi^) from WT and *Il1rl1*^*−/−*^ mice; n = 4 separate mice per group (C) Top upstream transcriptional regulators for transcriptomic changes from monocytes to pre-RPMs, or pre-RPMs to RPMs, in WT and *Il1rl1*^*−/−*^ mice, identified by Ingenuity pathway analysis. Note: the top regulators in each group were significant in both groups. (D) Top differentially expressed genes in pre-RPMs (CD11b^hi^ F4/80^lo^) from WT and *Il1rl1*^*−/−*^ mice; n = 4 separate mice per group. Please see also Table S2 and Figure S7.

**Figure 7:**
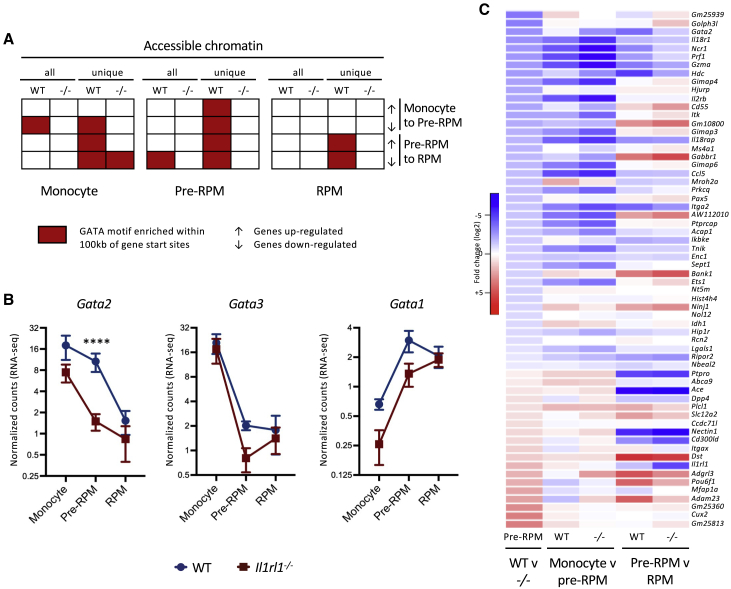
A GATA Switch May Underlie Changes in Gene Expression in the Development of Splenic Red Pulp Macrophages (RPMs) (A) Identification of GATA motifs in accessible chromatin of splenic monocytes (CD11b^hi^ F4/80^−^), pre-RPMs (CD11b^hi^ F4/80^lo^), and RPMs (CD11b^lo/−^ F4/80^hi^), within 100 kb of the start sites of genes differentially expressed between WT monocytes and WT pre-RPMs, or WT pre-RPMs and WT RPMs (both log2 fold change > 1, adjusted p value < 0.05). Motif enrichment was performed on accessible chromatin from each splenocyte population from WT and *Il1rl1*^*−/−*^ mice and also from accessible chromatin from each splenocyte population that was unique to WT or unique to *Il1rl1*^*−/−*^ mice; n = 4 separate mice per group. (B) Relative *Gata* gene expression (normalized counts, cpm) in splenic monocytes (CD11b^hi^ F4/80^−^), pre-RPMs (CD11b^hi^ F4/80^lo^), and RPMs (CD11b^lo/−^ F4/80^hi^) of WT (green) and *Il1rl1*^*−/−*^ mice (red); n = 4 separate mice per group. Data are represented as mean ± SEM. ^∗∗∗∗^ adjusted *p* (WT v *−/−*) < 0.001. (C) Heatmaps showing log2 fold changes of genes differentially expressed in WT and *Il1rl1*^*−/−*^ pre-RPMs (adjusted p value < 0.05) between splenic monocytes (CD11b^hi^ F4/80^−^) and pre-RPMs (CD11b^hi^ F4/80^lo^), or pre-RPMs (CD11b^hi^ F4/80^lo^) and RPMs (CD11b^lo/−^ F4/80^hi^), from WT and *Il1rl1*^*−/−*^ mice; n = 4 per group. Please see also Figure S7.

To provide further mechanistic insight into how IL1RL1-dependent IL-33 signaling promotes the development of monocyte-derived RPMs, we compared the transcriptomes of splenic monocytes, pre-RPMs, and RPMs from WT and IL1RL1-deficient mice (Figure 6; Table S1, Related to Figures 6 and 7 Differentially expressed genes in wild-type (wt) and Il1rl1−/− (ko) monocytes, adjusted p value<0.05, Table S2, Related to Figures 6 and 7 Differentially expressed genes in wild-type (wt) and Il1rl1−/− (ko) pre-RPM, adjusted p value<0.05, Table S3, Related to Figures 6 and 7 Differentially expressed genes in wild-type (wt) and Il1rl1−/− (ko) RPM, adjusted p value<0.05). The global gene expression profile of each cell type was very similar for WT and IL1RL1-deficient mice (Figures 6A and 6B, respectively). However, we identified a small number of differentially expressed genes between WT and IL1RL1-deficient splenocytes, with most differences in the pre-RPM cell population (Table S2), consistent with our prior observations that IL-33 promotes the development of RPMs from pre-RPMs *in vivo*.

We performed pathway analysis to identify potential transcriptional regulators of the genes that were differentially expressed between monocytes and pre-RPMs and between pre-RPMs and RPMs. Many of the same upstream regulators were identified in WT and in IL1RL1-deficient mice (Figure 6C). However, we noted that the transcription factors GATA2 and ETS1 (which is potentially regulated by GATA2; Linnemann et al., 2011), were implicated in the differentiation of monocytes to pre-RPMs and downregulated in IL1RL1-deficient pre-RPMs (Figure 6D; Table S2).

To further investigate the changes in gene expression that govern the differentiation of monocytes to RPMs, we assessed chromatin accessibility by ATAC-seq and then performed motif enrichment analysis to identify potentially important transcription factor binding sites in accessible regions of chromatin from splenic monocytes, pre-RPMs, and RPMs from WT and IL1RL1-deficient mice (Figure S7A; Table S4). There was a high degree of overlap in the accessible chromatin regions of each cell type between WT and IL1RL1-deficient mice (Figure S7A). However, GATA motifs were specifically enriched in monocytes in genes that were downregulated from monocytes to pre-RPMs, and in pre-RPMs in genes that were downregulated from pre-RPMs to RPMs, from WT but not IL1RL1-deficient mice (Figure 7A; Table S4). Indeed, accessible regions of chromatin that were unique to WT (i.e., inaccessible in IL1RL1-deficient mice) were enriched for GATA motifs within 100 kb of genes that were dynamically regulated in monocytes to pre-RPMs and in pre-RPMs to RPMs (Figure 7A). Thus, analysis of chromatin accessibility by ATAC-seq provided independent evidence for a role of GATA transcription factor signaling downstream of IL1RL1 in the development of monocyte-derived RPMs.

Expression of *Gata2* and *Gata3* was strongly downregulated, while *Gata1* expression was induced, in the differentiation of monocytes to RPMs, in both WT and IL1RL1-deficient mice (Figure 7B). This pattern of gene expression is consistent with a “GATA switch,” in which GATA1 replaces GATA2 at regulatory elements to affect changes in gene expression, as is well-documented in other pathways of hematopoiesis (Bresnick et al., 2010). GATA1 was identified, alongside GATA2, as another potential upstream regulator of the genes differentially expressed from monocytes to pre-RPMs, indicating that a GATA switch may indeed be involved in regulating this transition. However, there was a marked difference in the dynamics of *Gata2* expression in WT and IL1RL1-deficient mice (Figure 7B). In WT mice, *Gata2* expression was downregulated upon differentiation of pre-RPMs to RPMs; however, in IL1RL1-deficient mice, expression of *Gata2* was downregulated earlier, upon differentiation of monocytes to pre-RPMs (Figure 7B). It is likely that such a decrease in *Gata2* expression at this earlier stage would accentuate the GATA-switch-dependent changes in gene expression in the transition of monocytes to pre-RPMs, in IL1RL1-deficient mice compared to WT mice. Indeed, although the transcriptomes of WT and IL1RL1-deficient pre-RPMs were very similar, pathway analysis identified GATA2 and ETS1 as potential upstream regulators of most of the genes that were differentially expressed in WT versus IL1RL1-deficient pre-RPMs (Figure S7B). However, most compellingly, almost all of the genes that were differentially expressed between WT and IL1RL1-deficient pre-RPMs could be accounted for by accentuated changes in gene expression in the transition of monocytes to pre-RPMs in IL1RL1-deficient mice compared to WT mice (Figure 7C). Combined, these data support a role for IL1R1-dependent signaling in regulating the expression of GATA2, which is a key orchestrator of the transcriptional events in the development of monocyte-derived pre-RPMs that are capable of maturing to RPMs.

## Discussion

Local environmental cues are important in the specification of tissue-resident macrophages. This is the case for erythrocyte-derived heme that induces the expression of SPIC in monocytes, and contributes to the RPM differentiation program by establishing a precursor pre-RPM phenotype (Haldar et al., 2014).

Here, we have shown that IL-33 signaling via its receptor, IL1RL1, is required for the optimal generation of mature monocyte-derived RPMs. Mechanisms for how IL-33 regulates the functions of mononuclear cells have remained obscure despite roles in regulating mononuclear cell phenotypes (Andersson et al., 2018, Kiyomiya et al., 2015, McLaren et al., 2010, Vainchtein et al., 2018, Velickovic et al., 2015, Zaiss et al., 2011). Here, we implicated a role for IL-33 signaling in maintaining GATA2 activity that is important in regulating the changes in gene expression which, at least in part, instructed the transition of monocytes to pre-RPMs. By regulating GATA2 expression, IL-33 signaling influenced this transition to produce pre-RPMs that were competent to go on to fully differentiate to RPMs. In contrast, in the absence of IL-33 signaling, aberrant GATA2 expression led to terminal differentiation at a pre-RPM-like stage. Further resolution of the signaling pathways involved in the cooperation between heme and IL-33 for the development of mature RPMs will require additional investigations; however, our studies highlight a role for IL-33 combined with hemin in driving ERK1/2 activation, which culminates in fine-tuning of *Gata2* expression. The implication of ERK1/2 in the process was inferred from studies using a MEK inhibitor and will therefore require validation using genetic silencing.

GATA2 is an established regulator of myelopoiesis, with well-documented context and dose-dependent effects on stem and progenitor cell proliferation, quiescence, self-renewal, and differentiation (Kitajima et al., 2006, Ling et al., 2004, Nandakumar et al., 2015). *Gata2* expression is highest in stem and progenitor cells and downregulated during commitment in the monocyte lineage (Akashi et al., 2000). Further studies are required to ascertain the downstream effects of GATA2 that confer competency to pre-RPMs to continue to differentiate to RPMs, although our data implicate a potentially important role for a GATA switch, similar to those previously shown to regulate the expression of a number of GATA2 target genes, including *Gata*2 itself and some lineage-specific transcription factors such as the monocyte and macrophage lineage transcription factor PU.1 (also known as *Sfpi1*; Chou et al., 2009). Interactions between GATA1 and heme have previously been implicated in the control of erythropoiesis, notably in regulating the expression of genes involved in heme biosynthesis (Tanimura et al., 2016), and many of these genes were differentially expressed in the development of monocytes to RPMs (data not shown). Previous studies have also highlighted a role for a “definitive-hematopoiesis-specific” enhancer region, containing GATA, Ets, and AP-1 binding sites, 9.5 kb downstream of the *Gata2* transcriptional start site (Gao et al., 2013, Johnson et al., 2012). We found this region was accessible in WT but not IL1R1-deficient pre-RPMs (data not shown). We speculate that IL-33-mediated activation of ERK1/2 may regulate the recruitment of transcription factors to *Gata2* regulatory elements, such as the +9.5 enhancer, to regulate *Gata2* expression in the development of monocytes to pre-RPMs.

Here, we sought to clarify sources of IL-33 required for RPM development. We have discovered that native erythrocytes accumulate IL-33 and that erythrocyte-associated IL-33 is sufficient for RPM development *in vivo*. Erythrocytes have been shown to play a role in the storage and release of cytokines, although IL-33 was not investigated (Karsten et al., 2018). To date, evidence for expression of IL-33 on hematopoietic cells is very limited; however, it is noteworthy that platelets and *in vitro* cultured CD34^+^ erythroid progenitors were reported to express IL-33 (Takeda et al., 2016, Wei et al., 2015). Here, we have provided evidence for the existence of IL-33 in erythrocytes, using multiple techniques and supported by specificity controls, including samples from IL-33-deficient mice and highly purified erythrocyte preparations. In contrast to the detection of full-length IL-33 in murine erythrocytes, we found that IL-33 in human erythrocyte preparations was highly susceptible to proteolytic processing. Full-length IL-33 is biologically active; however, it is possible that proteolytic processing might regulate IL-33 activity (Lefrançais et al., 2014, Scott et al., 2018a). We could not rule out that this processing might have occurred during *ex vivo* handling, and further work is required to understand the nature of this processing and its biological relevance.

We explored mechanisms underlying the accumulation of IL-33 in erythrocytes. IL-33 localizes to the nucleus in nucleated cells, and it is unclear how IL-33 is stored in erythrocytes lacking a cell nucleus. We have found evidence for IL-33 gene transcription in bone-marrow-derived erythrocyte precursors consistent with studies in related hematopoietic cell precursors (Takeda et al., 2016, Wei et al., 2015). We also observed a partial role for IL1RL1 in acquiring and/or maintaining IL-33 in erythrocytes. However, overall, our data are inconsistent with IL-33 detected in mature erythrocytes being maintained on the cell surface by IL1RL1. The ELISA used to measure IL-33 in erythrocyte lysates does not detect IL-33 in complex with IL1RL1 (I.C.S. and E.S.C., unpublished data), and we were unable to detect endogenous IL-33 on the surface of erythrocytes or erythrocyte precursors (data not shown). We demonstrated the expression of IL1RL1 with binding activity on erythrocyte precursors in the bone marrow. Although the function of IL1RL1 during erythrocyte development is poorly understood, there is evidence for a role for IL-33 signaling in enucleation of erythroid precursors (Lopez-Yrigoyen et al., 2019). Still, additional studies will be required to elucidate mechanisms by which erythrocytes maintain a store of IL-33 in the absence of a nucleus and to further investigate the role of IL1RL1 in the bone marrow during the development of erythrocytes.

In conclusion, we have shown that IL-33 is unique in its local requirement for the development and specification of a tissue-resident macrophage phenotype, and in its cooperation with an essential biochemical compound (heme) generated by the same source, to fulfil its homeostatic function. This pathway is essential for the normal regulation of iron recycling. Similarly to its role as an alarmin in initiating immune responses during tissue damage, IL-33 appears to act as an alarmin during physiological damage of senescent erythrocytes, thereby instructing the differentiation of a specific subset of tissue-resident macrophages involved in the clearance of senescent erythrocytes and the recycling of their iron content. Beyond its role in iron homeostasis and the response to erythrocyte damage, IL-33 might also be involved in other immunological properties of RPMs, including the response to infections (Kurotaki et al., 2015).

## STAR★Methods

### Key Resources Table

**Table:** 

REAGENT or RESOURCE	SOURCE	IDENTIFIER
**Antibodies**		

Zombie Aqua Fixable Viability Kit	Thermo Fisher	Catalog number: L34965
Alexa Flour 700 anti-mouse CD11b	BD Bioscience	Catalog number: 557960; RRID: AB_396960
PE/Cy7 anti-mouse CD11c	BioLegend	Catalog number: 117317; RRID: AB_493569
Brilliant Violet 421 anti-mouse F4/80	BioLegend	Catalog number: 123131; RRID: AB_10901171
APC-eFluor 780 anti-mouse Ly6C	Thermo Fisher	Catalog number: 47-5932-82; RRID: AB_2573992
PerCP/Cy5.5 anti-mouse Ly-6G	BioLegend	Catalog number: 127653; RRID: AB_2616998
Brilliant Violet 650 anti-mouse NK-1.1	BioLegend	Catalog number: 108735; RRID: AB_11147949
Brilliant Violet 605 anti-mouse CD335	BioLegend	Catalog number: 137619; RRID: AB_2562452
FITC anti-mouse CD169	BioLegend	Catalog number: 142405; RRID: AB_2563106
APC anti-mouse CD209b	Thermo Fisher	Catalog number: 17-2093-82;RRID: AB_11151692
FITC anti-mouse T1/IL1RL1 (ST2)	MD Biosciences	Catalog number: 101001; RRID: AB_947549
FITC anti-mouse Ter119	BioLegend	Catalog number: 116205; RRID: AB_313706
PE-anti-mouse CD71	BioLegend	Catalog number: 113807; RRID: AB_313568
APC anti-mouse DYKDDDDK	BioLegend	Catalog number: 637307; RRID AB_2561496
Brilliant Violet 570 anti-mouse CD45.1	BioLegend	Catalog number: 110733; RRID: AB_10895765
Brilliant Violet 785 anti-mouse CD45.2	BioLegend	Catalog number: 109839; RRID: AB_2562604
APC anti-mouse CD45	BioLegend	Catalog number: 103111; RRID: AB_312976
anti-mouse IL-33 antibody	R&D systems	Catalog number: AF3626; RRID: AB_884269
anti-human IL-33 antibody	R&D systems	Catalog number: AF3625; RRID: AB_1151900
anti-human IL-33 antibody	BioRad	Catalog number: AHP1482; RRID: AB_2124144
anti-human IL-33 antibody	BioRad	Catalog number: AHP1626; RRID: AB_2124143
p44/42 MAPK (Erk1/2) Antibody	Cell Signaling	Catalog number: 9102; RRID: AB_330744
Phospho-p44/42 MAPK (Erk1/2) (Thr202/Tyr204) antibody	Cell Signaling	Catalog number: 9101; RRID: AB_331646
Phospho-NF-κB p65 antibody	Cell Signaling	Catalog number: 3033; RRID: AB_331284
anti-CD235a (Glycophorin A) MicroBeads, human	Miltenyi Biotec	Catalog number: 130-050-501
anti-Ter-119 MicroBeads, mouse	Miltenyi Biotec	Catalog number: 130-049-901

**Chemicals, Peptides, and Recombinant Proteins**		

Hemin	Sigma	Catalog number: 51280
U0126	Sigma	Catalog number: 662005
Recombinant Mouse IL-33 (carrier-free)	BioLegend	Catalog number: 580502
Recombinant Human IL-33 (carrier-free)	BioLegend	Catalog number: 581802
Soluble IL1RL1 (ST2)	AstraZeneca	Catalog number: N/A
Murine IgG1	AstraZeneca	Catalog number: N/A
Recombinant Mouse M-CSF (carrier-free)	BioLegend	Catalog number: 576402
Recombinant Human M-CSF (carrier-free)	BioLegend	Catalog number: 574802
Tn5 transposase	Illumina	Catalog number: 15027865
Transposase buffer	Illumina	Catalog number: 15027866
AMPure XP beads	Beckman Coulter	Catalog number: A63880

**Critical Commercial Assays**		

Human Phospho-Kinase Array Kit	R&D systems	Catalog number: ARY003B
PKH26 Red Fluorescent Cell Linker Kits	Sigma	Catalog number: MINI26
V-PLEX Plus Mouse IL-33 Kit	MesoScale Discovery	Catalog number: K152XBD-1
Human IL-33 Duoset ELISA kit	R&D systems	Catalog number: DY3625
SMARTer stranded total RNA-Seq v2 kit	Takara	Catalog number: 634412
RNEasy plus micro kit	Qiagen	Catalog number: 74034
Minelute PCR purification cleanup kit	Qiagen	Catalog number: 28004

**Tools**		

STAR 2.5.1	https://github.com/alexdobin/STAR/releases/tag/2.5.1b	RRID: SCR_015899
cutadapt 2.5	https://pypi.org/project/cutadapt/2.5/	RRID: SCR_011841
Python 3.6.2		RRID: SCR_008394
Subread-Feature Counts 1.6.2	https://sourceforge.net/projects/subread/files/subread-1.6.2/	RRID: SCR_009803
DESeq2 1.25.9	http://bioconductor.org/packages/release/bioc/html/DESeq2.html	RRID: SCR_015687
heatmap.2 function from the R Package gplots 3.0.1.1	https://cran.r-project.org/src/contrib/Archive/gplots/.	N/A
Ingenuity Systems Pathway analysis software	Qiagen	RRID: SCR_008653
TrimGalore	https://github.com/FelixKrueger/TrimGalore/releases/tag/0.6.4	RRID: SCR_011847
Bowtie2	https://sourceforge.net/projects/bowtie-bio/files/bowtie2/2.3.5.1	RRID: SCR_005476
Genrich	https://github.com/jsh58/Genrich#quick	N/A
BEDtools 2.29.0	https://github.com/arq5x/bedtools2/releases/tag/v2.29.0	RRID: SCR_006646
Meme-Chip	http://meme-suite.org/index.html	RRID: SCR_001783

**Data deposition**		

Sequencing data	GEO	GSE146782

### Lead Contact and Materials Availability

Lead author: Ziad Mallat, zm255@medschl.cam.ac.uk. Methods, including statements of data availability and any associated additional references are available in the online version of the paper.

### Experimental Model and Subject Details

#### Mice

All experiments were approved by the Home Office, UK. C57/Bl6 *Spic*^*igfp/igfp*^ reporter mice were kindly provided by Kenneth M. Murphy (Washington University, St Louis), BALB/c background of *Il1rl1*^*−/−*^ and *Il33*^*−/−*^ were from Andrew McKenzie and C57/Bl6 background of *Il1rl1*^*−/−*^ were from Padraic Fallon, C57/Bl6 *Myd88*^*−/−*^ mice were from Bernhard Ryffel and C57/Bl6 CD45.1 mice were originally from Jackson Labs. For the hemin (500 μg/200 μl) and IL-33 (1 μg/200 μl) *in vivo* experiment, the mice were injected intra-peritoneally once a day for 3 days. Control mice received PBS (200 μl) injections. For the ERK1/2 *in vivo* experiment, C57/Bl6 *Spic*^*igfp/igfp*^ reporter mice were treated with the MEK inhibitor U0126 or DMSO control 3 times per week for 6 weeks. U0126 solution was prepared in DMSO as previously described (Marampon et al., 2009). Two hundred microliters 50 μmol/kg were injected intra-peritoneally into each mouse. For the soluble IL1RL1 (ST2) *in vivo* experiment, C57/Bl6 *Spic*^*igfp/igfp*^ reporter mice were treated with soluble IL1RL1 or murine IgG1 control 200 μg/mouse 3 times per week for 6 weeks.

To reconstitute *Il33*^*−/−*^ mice with erythrocyte-associated IL-33, *Il33*^*−/−*^ mice received once a day for 3 days i.v. injections (200 μl) of 2x10^9^ erythrocytes from either WT or *Il33*^*−/−*^ mice.

### Method Details

#### Hemin preparation

Hemin was purchased from Sigma-Aldrich (51280, Sigma), and a stock solution was prepared at 25mg/mL in 0.15M NaCl containing 10% NH_4_OH and stored at −20°C. Hemin was used at a final concentration of 40uM for cell culture experiments and 500 μg/200 μL sterile 0.15M NaCl for i.p. injections *in vivo*, as previously described (Haldar et al., 2014).

#### Bone marrow transplants

CD45.1 mice were maintained overnight with Baytril before irradiation with two doses of 5.5 Gy (separated by 4 h) followed by reconstitution with 10^7^ bone marrow cells obtained from CD45.2 WT or *Il1rl1*^*−/−*^ mice. Donor-derived cells in spleen or liver (CD45.2^+^) were analyzed 10 weeks after transfer using flow cytometry. In other experiments, CD45.2 mice were lethally irradiated and injected intravenously (i.v.) with 10^7^ bone marrow cells containing 50% of CD45.2 WT or *Il1rl1*^*−/−*^ mice and 50% of CD45.1 mice.

#### Spleen, liver and serum iron analysis

Samples of the spleen (20-60 mg) and liver (40-90 mg) were digested (1 in 10) in 16% nitric acid, prepared by diluting a stock solution of high purity nitric acid (65% w/v p.a. plus; Sigma-Aldrich) with ultra-high purity water, in acid-cleaned 15 mL PTFE vials in an UltraWave Single Reaction Chamber Microwave Digestion System (Milestone Srl; Sorisole, Italy). The following digestion conditions were followed: 5 min ramp to 120°C, then 10 min ramp from 120°C to 230°C and maintained at 230°C for 15 min. Blank samples, containing just the acid mixture, were similarly prepared to check for background Fe contributions.

Digested samples were analyzed for total Fe by inductively coupled plasma optical emission spectrometry (Jobin Yvon Horiba Ultima 2C; Instrument SA, Longjumeau, France), equipped with a concentric micro-nebulizer and cyclonic spray chamber. A sample introduction pump speed of 10 rates/min, nebulizer flow rate of 0.72 mL/min and a plasma gas flow rate of 10 L/min was used with low-flow sample tubing. The 259.940 nm analytical line for Fe was used. All samples, including blank samples, were analyzed in a single batch with acid-based Fe standards (0-40 mg/L). Each sample was analyzed in triplicate and the average value was used. For serum measurements, 20 μl of serum was incubated with 20 μl of acid reagent for 5 min. Supernatant was mixed with 40 μl chromagen reagent, and absorbance at 535 nm was measured as described above.

#### Histochemical analysis

For Perl’s Prussian blue stain, tissues were fixed with 4% paraformaldehyde in 0.1 M phosphate buffer (pH 7.0), embedded in paraffin, and stained with Perl’s Prussian blue and pararosaniline (Sigma).

#### Differentiation of bone marrow-derived macrophages

Fresh bone marrow cells were used to generate BMDM. Cells were resuspended in 10 mL bone marrow differentiation media (RPMI1640 supplemented with 10% fetal bovine serum (GIBCO, cat. 12657-029), 100 U/mL penicillin, 100 μg/mL streptomycin, 2 mM L-glutamine and 20ng/mL of M-CSF(BioLegend)). Cells were seeded in Petri dishes (Corning Brand) and incubated at 37°C in a 5% CO_2_ atmosphere. Four days after seeding, an extra 10 mL of fresh differentiation media were added per plate and incubated for an additional 3 days.

#### Flow cytometry

Single-cell suspensions of bone marrow, spleen and liver were incubated with Fc block solution (eBioscience, clone 93), dilution of 1:200 in flow buffer (PBS, 1% BSA, 2 mM EDTA, 0.01% NaN3) for 10 min at 4°C. Cells were then stained with fluorophore-conjugated antibodies, dilution of 1:200 in flow buffer for 30 min at 4°C, prior to extensive wash and analyzed using an LSRII Fortessa (BD) flow cytometer. The following antibodies were used in the experiments, Zombie Aqua Fixable Viability Kit, Alexa Flour 700 anti-mouse CD11b (BD Bioscience MI170), PE/Cy7 anti-mouse CD11c (BioLegend N418), Brilliant Violet 421 anti-mouse F4/80 (BioLegend BM8), APC-eFluor 780 anti-mouse Ly6C (eBioscience HK1.4), PerCP/Cy5.5 anti-mouse Ly-6G (BioLegend 1A8), Brilliant Violet 650 anti-mouse NK-1.1 (BioLegend PK136), Brilliant Violet 605 anti-mouse CD335 (BioLegend 29A1.4), FITC anti-mouse CD169 (BioLegend 3D6.112), APC anti-mouse CD209b (eBioscience eBio22D1), FITC anti-mouse T1/IL1RL1 (ST2) (mdbioproducts DJ8), FITC anti-mouse Ter119 (BioLegend Ter119), PE anti-mouse CD71 (BioLegend RI7217), APC anti-mouse DYKDDDDK (BioLegend L5), Brilliant Violet 570 anti-mouse CD45.1 (BioLegend A20) and Brilliant Violet 785 anti-mouse CD45.2 (BioLegend 104), APC anti-mouse CD45 (BioLegend 30-F11). Cell analysis was done using BD FACSDiva v8.01 Software and figure displayed dot plots and histograms were obtained using FlowJo v10.5 software (FlowJo, LLC).

#### Cytokine quantification

We used MesoScale Discovery IL-33 V-Plex assay kit (product code K152XBD-1) and human IL-33 Duoset ELISA kit (DY3625, R&D systems) according to manufacturer’s instructions. Measurements were performed blindly by the Core Biochemical Assay lab at Addenbrookes Hospital, the University of Cambridge, Cambridge, UK.

#### Quantitative RT-PCR

For gene expression analysis, RNA from sorted RPM, Pre-RPM and Monocytes were isolated using an RNAeasy mini kit (QIAGEN). RT-PCR was performed using a QuantiTect Reverse Transcription kit (QIAGEN). Real-time PCR was performed on 5 μL cDNA product (diluted 10 to 20 times) using SYBR Green qPCR mix (Eurogentec) on a Roche Lightcycler. Primer sequences are: human *SPIC* forward 5′-ACGGTAATTAACAGTGCTGCG-3′ and reverse 5′-GCTGGAGAAGAGTGGGTTGT-3′, human *HMOX1* forward 5′-TAGAAGAGGCCAAGACTGCG-3′ and reverse 5′- GGGCAGAATCTTGCACTTTGTT-3′, human *TREML4* forward 5′- AGACCAGGAAATCAAGAGCCC-3′ and reverse 5′-AAACCTCGTCACTGCTGTCC-3′, human *VCAM1* forward 5′-GTTTGCAGCTTCTCAAGCTTTT-3′ and reverse 5′-AGATGTGGTCCCCTCATTCG −3′, human *LCN2* forward 5′-GCCCTGAAATCATGCCCCTA-3′ and reverse 5′-TCCCCTGGAATTGGTTGTCC-3′, human *BACH1* forward 5′-TCGCGTAAGAAAAGCCGAG-3′ and reverse 5′-CATCAACCATATTGTGCGAGGC-3′, human *IL1RL1* forward 5′- CCCTCTGTCTTTCAGTTTGGTTGA-3′ and reverse 5′- ACGACAGTGAAGGTCACCAC-3′. Mouse *Spic* forward 5′-ACTGGAGAGGTGTAACAAATGGT-3′ and reverse 5′-CAAACAGCCGAAGCTTTCTCC-3′, mouse *Treml4* forward 5′-AAGCACAGCCACCATCTTTATG-3′ and reverse 5′-GCACACAGAAAACTGACAGCA-3′, mouse *Bach1* forward 5′-CTCTGAGACGGACACGGAAG-3′ and reverse 5′-CCTTCTGCGGATGTCATGGA-3′, mouse *Itgam* (Cd11b) forward 5′-GTGAGGTCTAAGACAGAGACCAA-3′ and reverse 5′-TGCCGCTTGAAAAAGCCAAG-3′, mouse *Hmox1* forward, 5′-GCTAGCCTGGTGCAAGATACT-3′ and reverse 5′-TGGGGGCCAGTATTGCATTT-3′, mouse *Il1rl1* forward 5′-CCAGCCCTTCATCTGGGTATC-3′ and reverse 5′-TGGCAATGGCACAGGATAGT-3′, mouse *Lcn2* forward 5′-CTGTCCCAATCGACCAGTGT-3′ and reverse 5′-CCAGCTCCCTCAATGGTGTT-3′, mouse *Adgre1* (F4/80) forward 5′-AATCGCTGCTGGTTGAATACAG-3′ and reverse 5′-CCAGGCAAGGAGGACAGAGTT-3′, mouse *Vcam1* forward 5′-CCGGCATATACGAGTGTGAA-3′ and reverse 5′-GATGCGCAGTAGAGTGCAAG-3′.

These experiments were independently repeated three times and each treatment consisted of triplicate samples.

#### PKH26-staining of Mouse RBCs and analysis of PKH26-stained cells

Anti-mouse CD47 Fab antibody treated RBCs were stained with PKH26 (Sigma) according to the manufacturer’s protocol. Briefly, 250 μL of RBCs were mixed with 2.25 mL diluent C from the PKH26 cell linker kit and incubated with 2 μM PKH26 dye for 5 min at room temperature. The staining reaction was stopped by the addition of 2.5 mL FBS for 1 min, followed by further dilution with 5 mL PBS. Cells were then washed three times with PBS and resuspended at 1x10^10^/200 μL for intravenous injection. 24 h after i.v. injection, spleens were collected and single-cell suspensions were prepared by mechanical disruption. Following incubation in Fc block, splenocytes were labeled with antibodies against Ly6G, CD11b and F4/80 and analyzed by flow cytometry (Fortessa) for PKH26+ cells in splenic monocytes (CD11b^hi^ F4/80^-^) or pre-RPM (CD11b^hi^ F4/80^lo^) or RPM (CD11b^lo/-^ F4/80^hi^) or neutrophil (Ly6G^hi^CD11b^+^) population.

#### Crude RBC lysates preparation

Human or mouse whole blood was collected in EDTA-treated blood collection tubes, followed by centrifugation at 400 g for 10 min. The RBC were transferred to new tubes and washed twice in PBS. The samples were incubated with RBC lysis buffer at room temperature for 10 min. After centrifugation at 13,000 g for 15 min at 4°C, the supernatants were passed through Amicon ultra (50 kDa) cutoff filter to remove haemoglobin (64 kDa), followed by a centrifugation at 7,000 g for 20 min at 4°C.

#### Enriched human RBC lysates preparation

The positive selection of human erythrocytes from fresh human whole blood was performed using anti-CD235a (Glycophorin A)-conjugated microbeads and MACS LS columns (Miltenyi Biotec) according to manufacturer’s instructions. Enriched erythrocyte preparations (2e8 cells/mL) were lysed in PBS/0.1% Triton X-100 at 4°C and lysates snap frozen on dry ice.

#### SDS-PAGE and western blot analysis

Samples were analyzed by SDS-PAGE on NuPAGE Novex 4%–12% or 12% Bis-Tris mini gels (Invitrogen) with MOPS running buffer (Invitrogen) according to manufacturer’s instructions under reducing conditions. All samples were reduced by heating to 95°C for 3 min in SDS-PAGE buffer containing 2% beta-mercaptoethanol. Proteins were transferred to nitrocellulose membranes (Invitrogen) and detected by western blotting. IL-33 was detected with an anti-mouse IL-33 antibody (R&D systems AF3626) and anti-human IL-33 antibodies (R&D systems AF3625 and BioRad AHP1482, AHP1626). Immunoreactive proteins were identified with HRP-conjugated secondary antibodies (R&D systems HAF008, 005 and 109) and Supersignal West Femto substrate (Pierce 34095) and visualized on LI-COR cDigit.

#### RNA-sequencing analysis in splenocytes

RNA was extracted from freshly sorted splenic monocytes, pre-RPM and RPM, using the RNEasy plus micro kit (QIAGEN). RNA libraries were prepared using the SMARTer stranded total RNA-Seq v2 kit (Takara) and sequenced using Illumina NovaSeq (2x50bp), obtaining approximately 20 million reads per sample.

#### ATAC-sequencing analysis in splenocytes

ATAC-seq was performed according to the published Omni-ATAC protocol (Corces et al., 2017). Briefly, nuclei were purified from 5x10^4^ freshly sorted splenic monocytes, pre-RPM and RPM. Transposition reactions were performed in 50 μL using Tn5 transposase (Illumina) for 30 min at 37°C. Libraries were prepared using the recommended Nextera barcodes (Corces et al., 2017) and the number of PCR amplification cycles was determined empirically as the number of cycles required to generate 25% of the maximum of a 5 μL aliquot, as recommended (Corces et al., 2017). Libraries were cleaned up using the Minelute PCR purification cleanup kit (QIAGEN) followed by AMPure XP beads (Beckman Coulter) and quantified using quantitative PCR (Kapa Biosystems). 5nM libraries were multiplexed and sequenced using Illumina NovaSeq (2x50bp), obtaining approximately 80 million reads per barcoded sample.

#### Differential gene expression (DGE) analysis

Reads were mapped to Ensembl/GRCm38.95 using STAR (v2.5.1) (https://github.com/alexdobin/STAR/releases/tag/2.5.1b) with default parameters, trimmed with cutadapt 2.5 (https://pypi.org/project/cutadapt/2.5/) under Python 3.6.2 (parameters:–nextseq-trim = 20 -m 15 for the R1 reads and–nextseq-trim = 20 -m 15 -u 3 for the R2 reads) and counted using Subread-Feature Counts (v1.6.2) (https://sourceforge.net/projects/subread/files/subread-1.6.2/). DGE analysis was performed using DESeq2 (v1.25.9) (http://bioconductor.org/packages/release/bioc/html/DESeq2.html). Heatmaps were generated using the heatmap.2 function from the R Package gplots version 3.0.1.1 (https://cran.r-project.org/src/contrib/Archive/gplots/). Lists of differentially expressed genes were submitted to Pathway analysis using Ingenuity Systems Pathway analysis software (QIAGEN).

#### ATAC peak calling & Meme

Reads were quality trimmed using TrimGalore (https://github.com/FelixKrueger/TrimGalore/releases/tag/0.6.4) with a quality Phred score cutoff of 20 and a minimum required sequence length for both reads before a sequence pair gets removed set to 20 bp. Trimmed reads were mapped to Ensembl/GRCm38.95 using Bowtie2 (https://sourceforge.net/projects/bowtie-bio/files/bowtie2/2.3.5.1/) with standard parameters. Peaks were called using Genrich (https://github.com/jsh58/Genrich#quick) and excluding mitochondrial reads. Differential peak analysis was performed using BEDtools 2.29.0 (https://github.com/arq5x/bedtools2/releases/tag/v2.29.0). Motif enrichment was performed using Meme-Chip (Ma et al., 2014) with default parameters.

### Quantification and Statistical Analysis

Data are represented as mean ± SEM. Differences between values were examined using the parametric two-tailed unpaired Student’s t test or two-way ANOVA.

### Data and Code Availability

All sequencing data have been deposited in the National Centre for Biotechnology Information Gene Expression Omnibus (GEO). The accession number for the sequencing data reported in this paper is GEO: GSE146782. All other relevant data and scripts are available on request.

## Supplemental Information

Document S1. Figures S1–S7

Table S1, Related to Figures 6 and 7 Differentially expressed genes in wild-type (wt) and Il1rl1−/− (ko) monocytes, adjusted p value<0.05

Table S2, Related to Figures 6 and 7 Differentially expressed genes in wild-type (wt) and Il1rl1−/− (ko) pre-RPM, adjusted p value<0.05 .

Table S3, Related to Figures 6 and 7 Differentially expressed genes in wild-type (wt) and Il1rl1−/− (ko) RPM, adjusted p value<0.05 .

Table S4, Related to Figure 7 Motifs significantly enriched in accessible chromatin of splenocytes from wild-type (wt) and Il1rl1−/− (ko) mice .

Document S2. Article plus Supplemental Information
