# Supplementary material for: Interleukin-33 Signaling Controls the Development of Iron-Recycling Macrophages
Source: Immunity. 2020 May 19;52(5):782–793.e5. doi: 10.1016/j.immuni.2020.03.006 (PMC7237885; doi:10.1016/j.immuni.2020.03.006)
Supplement: Document S1. Figures S1–S7 [file mmc1.pdf]

Immunity, Volume *52*

Supplemental Information

Interleukin-33 Signaling Controls the Development of Iron-Recycling Macrophages

Yuning Lu, Gemma Basatemur, Ian C. Scott, Davide Chiarugi, Marc Clement, James Harrison, Ravin Jugdaohsingh, Xian Yu, Stephen A. Newland, Helen E. Jolin, Xuan Li, Xiao Chen, Monika Szymanska, Guttorm Haraldsen, Gaby Palmer, Padraic G. Fallon, E. Suzanne Cohen, Andrew N.J. McKenzie, and Ziad Mallat

Figure S1

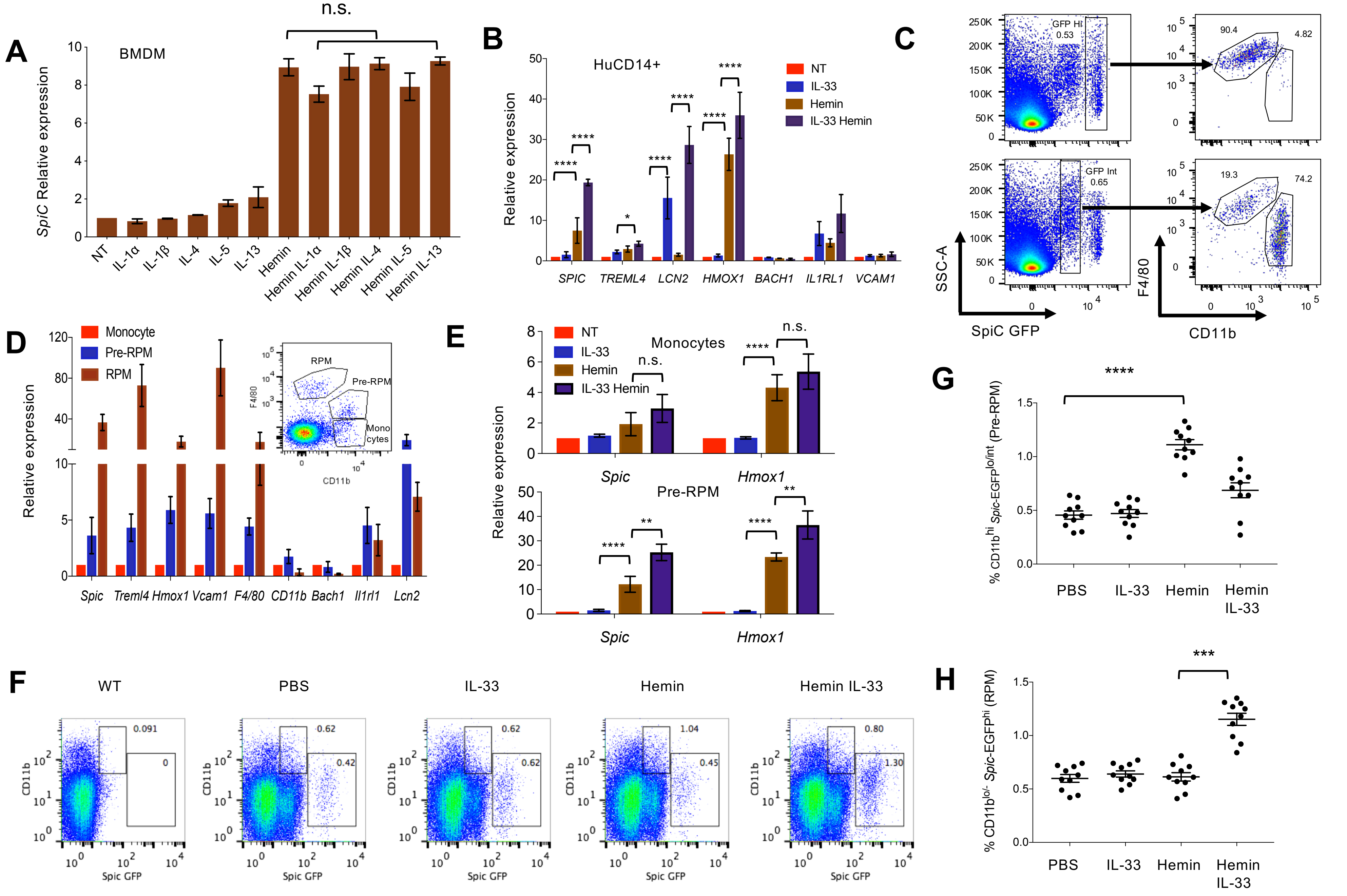

A
BMDM
SpiC Relative expression
n.s.
NT
IL-1α
IL-1β
IL-4
IL-5
IL-13
Hemin
Hemin IL-1α
Hemin IL-1β
Hemin IL-4
Hemin IL-5
Hemin IL-13
B
HuCD14+
Relative expression
NT
IL-33
Hemin
IL-33 Hemin
SPIC
TREML4
LCN2
HMOX1
BACH1
IL1RL1
VCAM1
C
GFP Hi
0.53
90.4
4.82
GFP Int
0.65
19.3
74.2
SSC-A
SpiC GFP
F4/80
CD11b
D
Monocyte
Pre-RPM
RPM
Relative expression
RPM
Pre-RPM
Mono cytes
F4/80
CD11b
Spic
Treml4
Hmox1
Vcam1
F4/80
CD11b
Bach1
Il1rl1
Lcn2
E
NT
IL-33
Hemin
IL-33 Hemin
Monocytes
n.s.
Relative expression
Spic
Hmox1
Pre-RPM
F
WT
PBS
IL-33
Hemin
Hemin IL-33
0.091
0
0.62
0.42
0.62
0.62
1.04
0.45
0.80
1.30
CD11b
Spic GFP
G
% CD11b^hi Spic-EGFP^lo/int (Pre-RPM)
PBS
IL-33
Hemin
Hemin IL-33
H
% CD11b^lo/- Spic-EGFP^hi (RPM)
PBS
IL-33
Hemin
Hemin IL-33

Figure S1 related to Figure 1. IL-33 up-regulates hemin-induced *Spic* expression and promotes the development of a red pulp macrophage (RPM) phenotype *in vitro* and *in vivo*. **A**, *Spic* mRNA expression in mouse bone marrow-derived (BMDM) macrophages stimulated *in vitro* for 4 days with individual cytokines (10ng/mL), hemin (40μM), or a combination of hemin and individual cytokines, compared to no treatment (NT). Data are representative of 3 independent experiments. **B**, Human macrophages were developed in culture from isolated blood CD14+ monocytes (HuCD14+) and were treated *in vitro* for 4 days with hemin, IL-33, or a combination of hemin + IL-33, and were assessed for mRNA expression of *Spic* and other typical red pulp macrophage-associated genes, including *Treml4*, *Lcn2*, *Hmox1*, *Il1rl1* and *Vcam1*. Data are representative of 3 independent experiments. **C**, Representative examples of flow cytometry staining for *Spic*-EGFP, CD11b and F4/80 in splenocytes of C57/Bl6 $Spic^{igfp/igfp}$ reporter mice. Splenic RPM are $CD11b^{lo/-}$ $F4/80^{hi}$ *Spic*-$EGFP^{hi}$, whereas most *Spic*-$EGFP^{int}$ cells are $CD11b^{hi}$ $F4/80^{lo/-}$ pre-RPM and monocytes. Data are representative of more than 3 independent experiments. **D**, Splenic monocytes ($CD11b^{hi}$ $F4/80^{-}$), pre-RPM ($CD11b^{hi}$ $F4/80^{lo}$) and RPM ($CD11b^{lo/-}$ $F4/80^{hi}$) were cell-sorted by flow cytometry and were assessed for mRNA expression of *Spic* and other typical RPM-associated genes. Data are representative of 3 pooled mice per group. **E**, Cell-sorted (flow cytometry) splenic monocytes ($CD11b^{hi}$ $F4/80^{-}$) and pre-RPM ($CD11b^{hi}$ $F4/80^{lo}$) were treated *in vitro* for 4 days with hemin (40 μM), IL-33 (10 ng/mL), or a combination of hemin + IL-33, and compared to untreated cells for the expression of *Spic* and *Hmox1*. Data are representative of 3 pooled mice per group. **F**, C57/Bl6 $Spic^{igfp/igfp}$ reporter mice were injected intraperitoneally once a day for 4 days with either phosphate buffered saline (PBS), IL-33 (1μg), hemin (500μg), or IL-33 + hemin, and spleens were assayed by flow cytometry for splenic RPM ($CD11b^{lo/-}$ *Spic*-$EGFP^{hi}$) and pre-RPM ($CD11b^{hi}$ *Spic*-$EGFP^{lo/int}$). Quantification of (**G**) pre-RPM and (**H**) RPM (percentages among $CD11c^{low}$ $Ly6G^{low}$ $NK1.1^{low}$ $SSC\text{-}A^{low}$ cells). Each dot in **G** and **H** represents a separate mouse. ****P<0.0001; ***P<0.001.

Figure S2

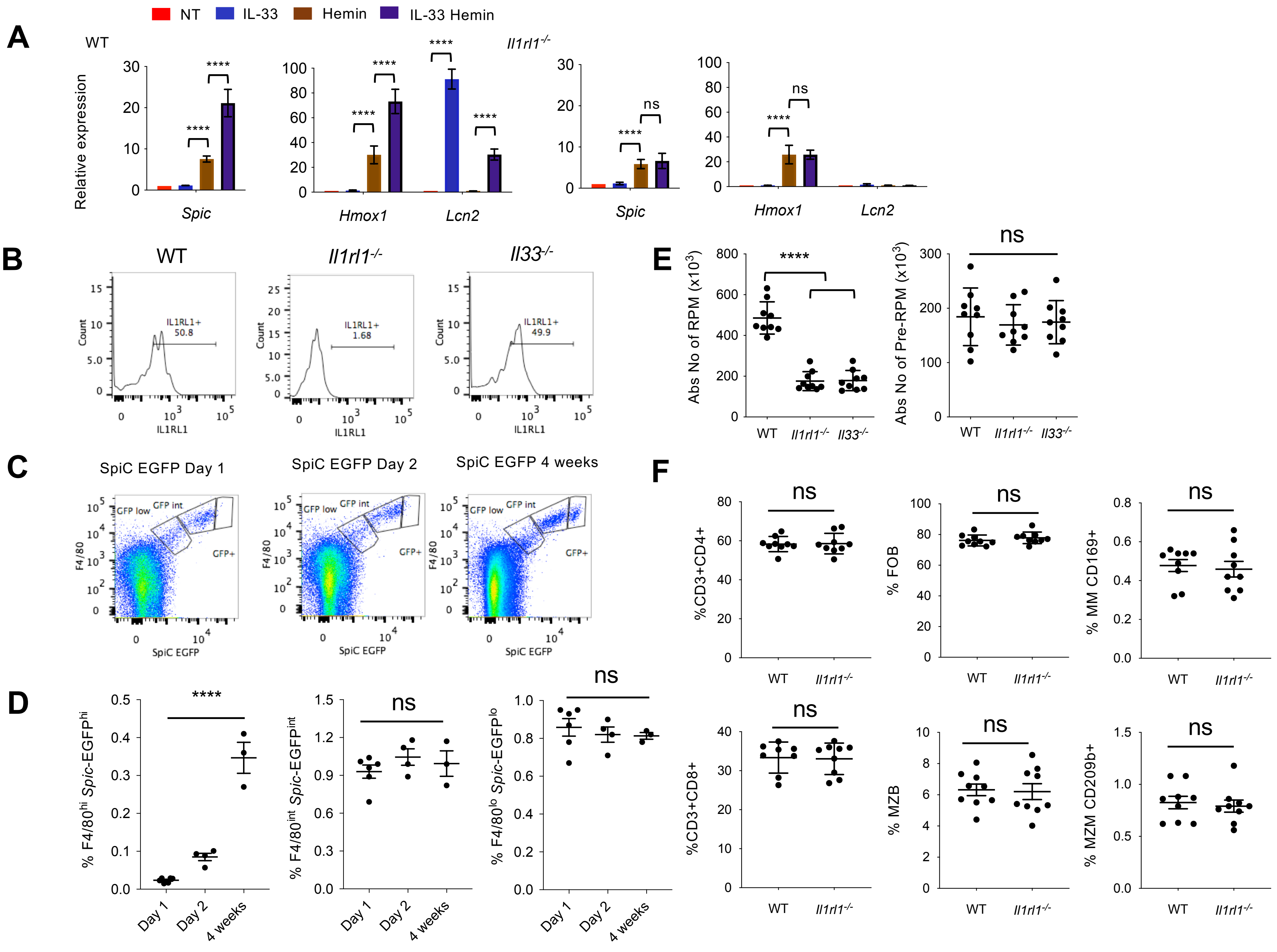

NT
IL-33
Hemin
IL-33 Hemin
A
WT
Relative expression
Spic
Hmox1
Lcn2
Il1rl1-/-
ns
B
WT
Il1rl1-/-
Il33-/-
IL1RL1+ 50.8
IL1RL1+ 1.68
IL1RL1+ 49.9
Count
IL1RL1
C
SpiC EGFP Day 1
SpiC EGFP Day 2
SpiC EGFP 4 weeks
GFP low
GFP int
GFP+
F4/80
SpiC EGFP
D
% F4/80hi Spic-EGFPhi
% F4/80int Spic-EGFPint
% F4/80lo Spic-EGFPlo
Day 1
Day 2
4 weeks
E
Abs No of RPM (x10³)
Abs No of Pre-RPM (x10³)
WT
Il1rl1-/-
Il33-/-
F
%CD3+CD4+
% FOB
% MM CD169+
%CD3+CD8+
% MZB
% MZM CD209b+

Figure S2 related to Figure 2. IL1RL1 (ST2) signalling controls the development of splenic red pulp macrophages (RPM). **A**, *Spic*, *Hmox1* and *Lcn2* mRNA expression in wild type (WT) and *Il1rl1*$^{-/-}$ mouse bone marrow-derived macrophages stimulated *in vitro* for 4 days with IL-33 (10 ng/mL), hemin (40 μM), or a combination of hemin and IL-33, compared to untreated cells (NT). Data are representative of 5 independent experiments per group. **B**, Representative examples of IL1RL1 (ST2) expression in splenic RPM (CD11b$^{lo/—}$ F4/80^{hi}) of wild-type (WT), *Il1rl1*$^{-/-}$ (negative control), and *Il33*$^{-/-}$ mice. **C**, Representative examples of *Spic*-EGFP and F4/80 expression by flow cytometry in spleens of neonates (Day 1 and Day 2) and young (4 weeks old) mice. **D**, Quantification of F4/80^{hi} *Spic*-EGFP^{hi}, F4/80^{int} *Spic*-EGFP^{int} and F4/80^{lo} *Spic*-EGFP^{lo} cells (percentages among CD11c^{low} Ly6G^{low} NK1.1^{low} SSC-A^{low} cells). **E,** Quantification of RPM and pre-RPM (absolute numbers) in spleens of 6 weeks and 42 weeks old wild type (WT), *Il33*$^{-/-}$, and *Il1rl1*$^{-/-}$ mice. **F,** Quantification of other immune cell types in spleens of 6 weeks old wild type (WT) and *Il1rl1*$^{-/-}$ mice. FOB= Follicular B cells; MZB= marginal zone B cells; MM= Metallophilic macrophages; MZM= Marginal zone macrophages. Each dot in **D, E** and **F** represents a separate mouse. ****P<0.0001.

Figure S3

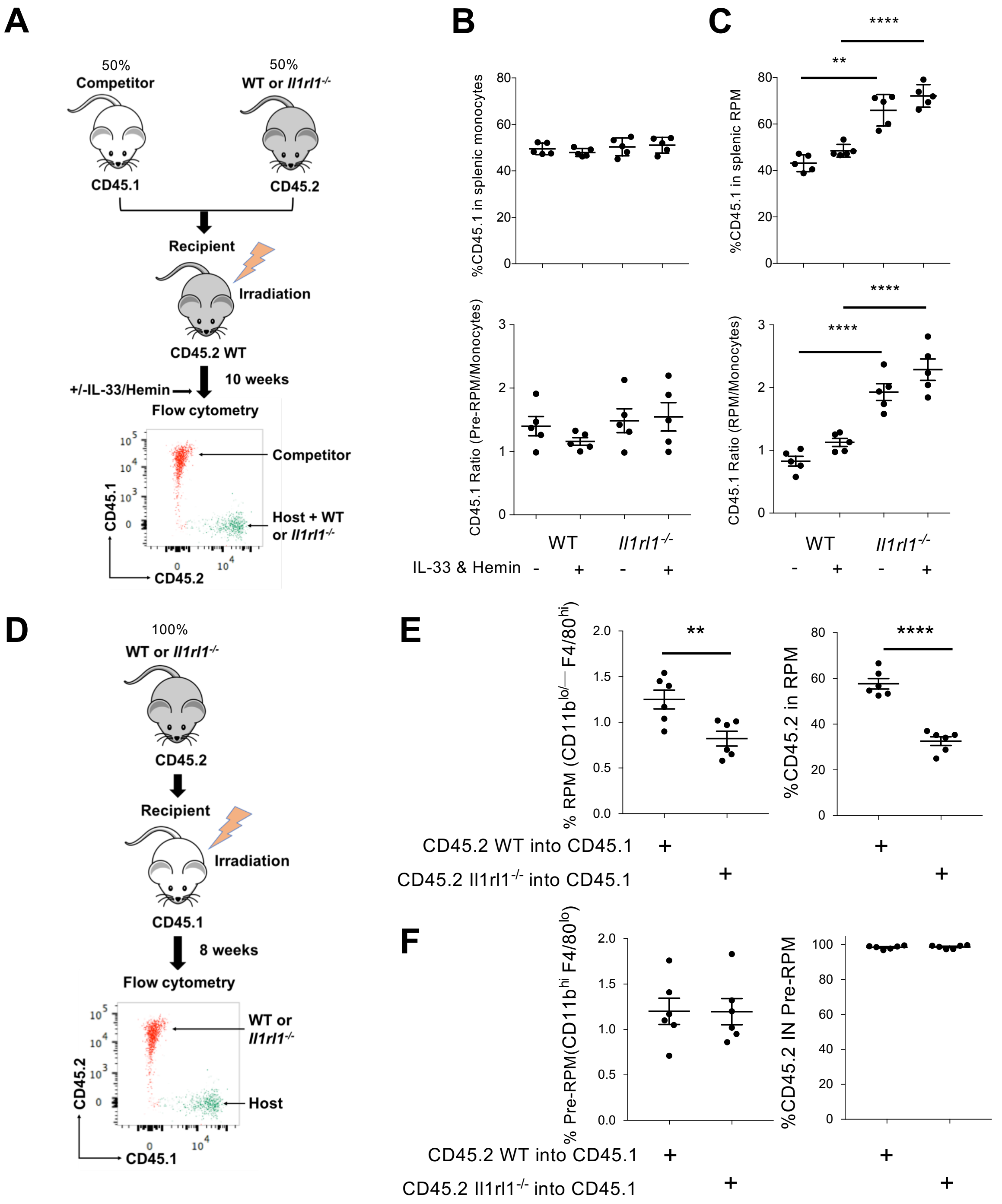
A
50%
Competitor
50%
WT or Il1rl1-/-
CD45.1
CD45.2
Recipient
Irradiation
CD45.2 WT
+/-IL-33/Hemin
10 weeks
Flow cytometry
Competitor
Host + WT or Il1rl1-/-
CD45.1
CD45.2
B
%CD45.1 in splenic monocytes
CD45.1 Ratio (Pre-RPM/Monocytes)
WT
Il1rl1-/-
IL-33 & Hemin
C
%CD45.1 in splenic RPM
CD45.1 Ratio (RPM/Monocytes)
D
100%
WT or Il1rl1-/-
CD45.2
Recipient
Irradiation
CD45.1
8 weeks
Flow cytometry
WT or Il1rl1-/-
Host
E
% RPM (CD11b lo/− F4/80 hi)
%CD45.2 in RPM
CD45.2 WT into CD45.1
CD45.2 Il1rl1-/- into CD45.1
F
% Pre-RPM(CD11b hi F4/80 lo)
%CD45.2 IN Pre-RPM

Figure S3 related to Figure 2. Cell-autonomous requirement of IL1RL1 expression in bone marrow-derived monocytes during the development of splenic red pulp macrophages (RPM). **A**, Representative scheme of lethal irradiation and reconstitution of CD45.2 WT mice with either 50% CD45.1 + 50% CD45.2 WT or 50% CD45.1 + 50% CD45.2 *Il1rl1*$^{-/-}$ bone marrow. 10 weeks later, mice were injected intraperitoneally once a day for 3 days with either phosphate buffered saline (PBS), or IL-33+hemin (see Methods), before they were assayed for CD45.1 and CD45.2 positive cells by flow cytometry. **B**, **C**, Quantification of CD45.1 positive cells among splenic monocytes (CD11b^{hi} F4/80^{-}), pre-RPM (CD11b^{hi} F4/80^{lo}) and RPM (CD11b$^{lo/-}$ F4/80^{hi}) by flow cytometry. Each dot represents a separate mouse. **P<0.01, ****P<0.001. **D-F**, CD45.1 wild-type (WT) mice were lethally irradiated and reconstituted with either 100% CD45.2 WT or 100% CD45.2 *Il1rl1*$^{-/-}$ (**D**) bone marrow. 8 weeks after bone marrow reconstitution, spleens were assayed for CD45.1 and CD45.2 positive RPM (CD11b$^{lo/-}$ F4/80^{hi}) and pre-RPM (CD11b^{hi} F4/80^{lo}) by flow cytometry. Quantifications (percentages and absolute numbers) are shown in (**E**) for RPM, and in (**F**) for pre-RPM. Each dot in **E** and **F** represents a separate mouse. **P<0.001. ****P<0.0001.

Figure S4

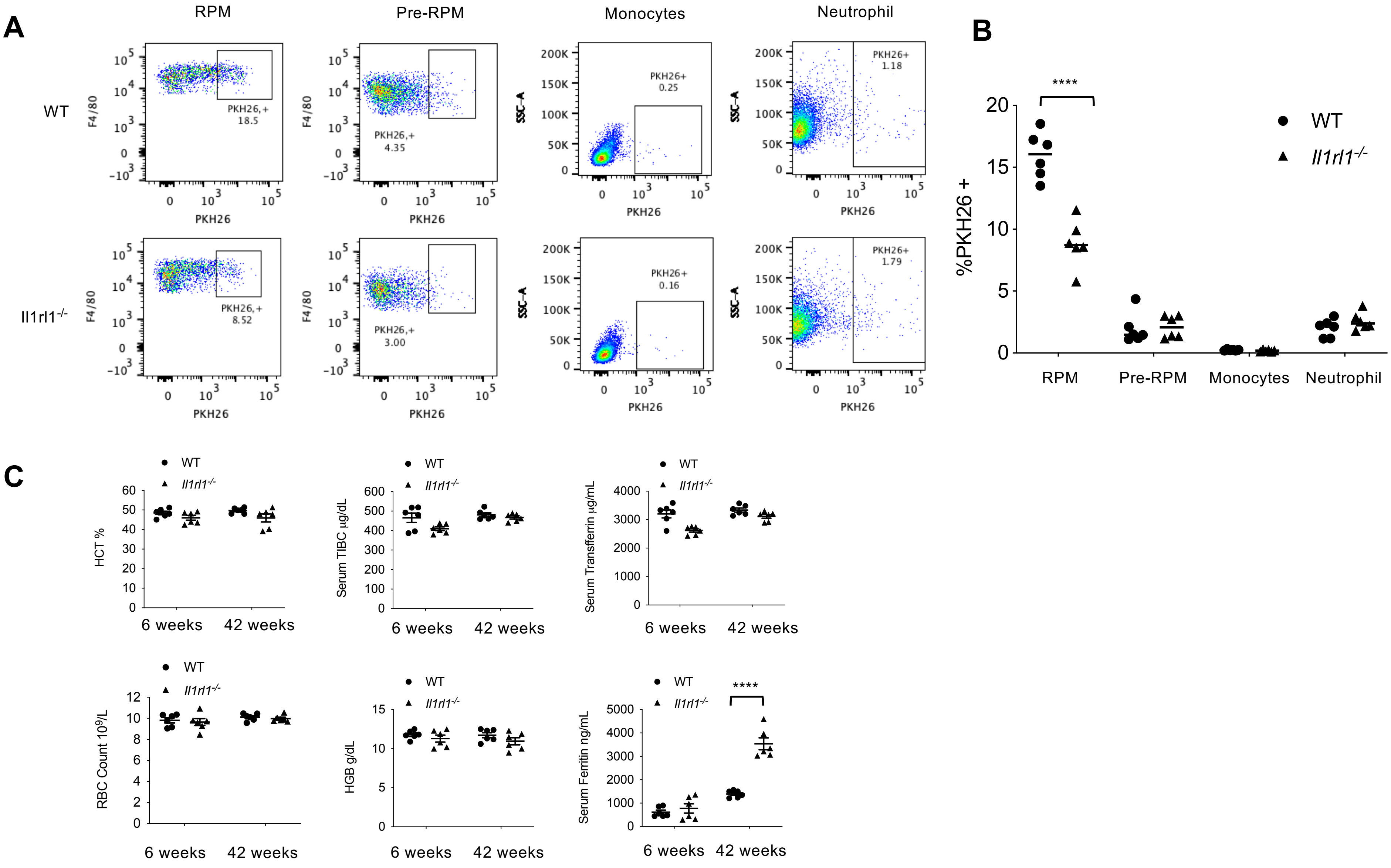

A
RPM
Pre-RPM
Monocytes
Neutrophil
WT
Il1rl1-/-
F4/80
SSC-A
PKH26
PKH26,+ 18.5
PKH26,+ 4.35
PKH26+ 0.25
PKH26+ 1.18
PKH26,+ 8.52
PKH26,+ 3.00
PKH26+ 0.16
PKH26+ 1.79
B
%PKH26 +
****
WT
Il1rl1-/-
RPM
Pre-RPM
Monocytes
Neutrophil
C
HCT %
Serum TIBC µg/dL
Serum Transferrin µg/mL
RBC Count 10^9/L
HGB g/dL
Serum Ferritin ng/mL
6 weeks
42 weeks
****

Figure S4 related to Figure 3. Impact of IL1RL1 (ST2) signalling on the function of splenic red pulp macrophages (RPM) . Representative examples (6 mice per group) of flow cytometry staining (**A**) and quantification (**B**) of uptake of PKH26-labelled red blood cells by splenic RPM, pre-RPM, monocytes and neutrophils after injection into WT and *Il1rl1*$^{-/-}$ mice. **C**, Quantification of hematocrit (HCT), red blood cell (RBC) counts, serum (TIBC), hemoglobin (HGB), transferrin and ferritin in young (6 weeks) and old (42 weeks) WT and *Il1rl1*$^{-/-}$ mice. Each dot in **B** and **C** represents a separate mouse. ****P<0.001.

Figure S5

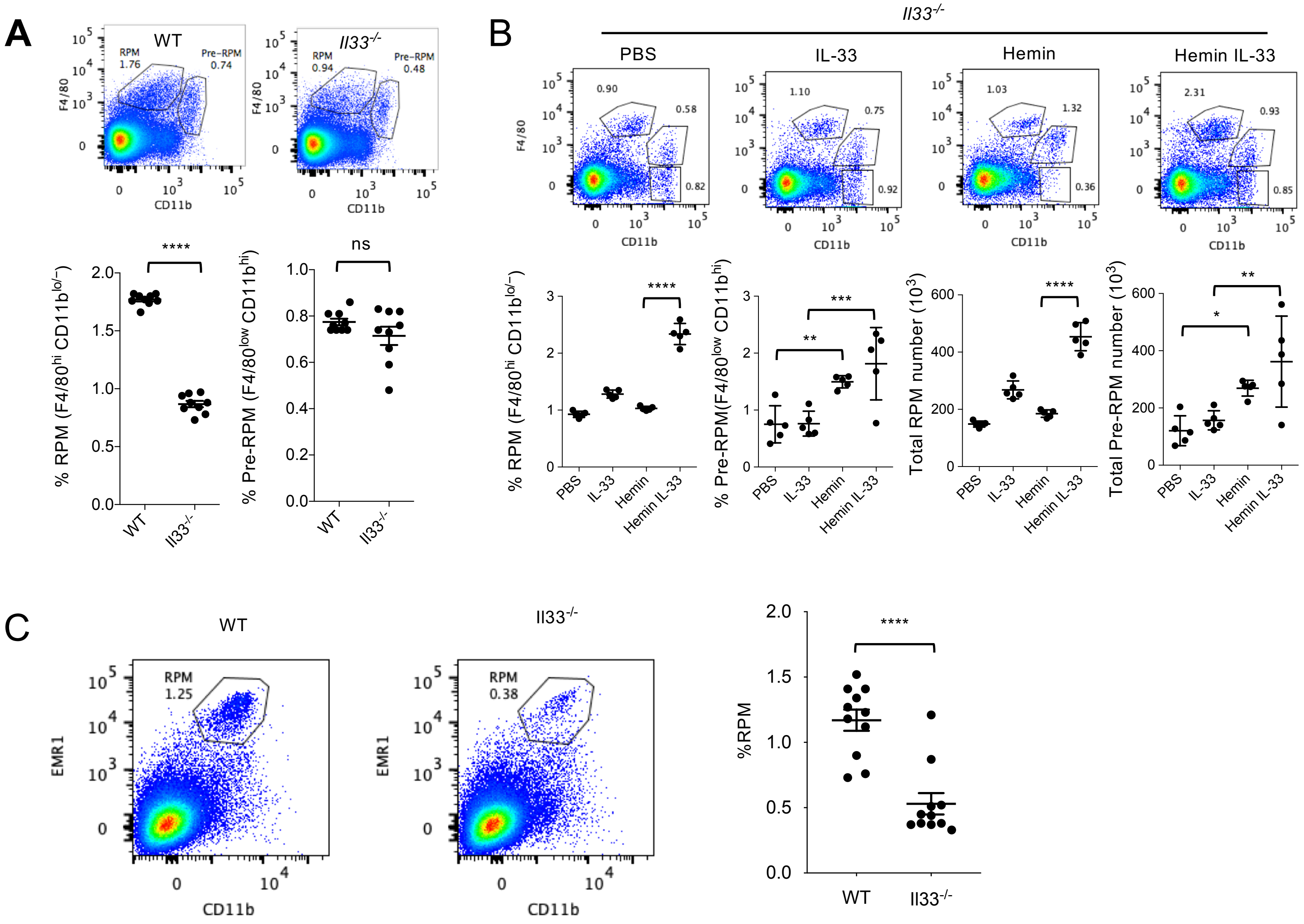
A
WT
Il33-/-
RPM 1.76
Pre-RPM 0.74
RPM 0.94
Pre-RPM 0.48
F4/80
CD11b
% RPM (F4/80hi CD11blo/–)
% Pre-RPM (F4/80low CD11bhi)
****
ns
B
Il33-/-
PBS
IL-33
Hemin
Hemin IL-33
0.90
0.58
0.82
1.10
0.75
0.92
1.03
1.32
0.36
2.31
0.93
0.85
Total RPM number (10^3)
Total Pre-RPM number (10^3)
C
RPM 1.25
RPM 0.38
EMR1
%RPM

Figure S5 related to Figure 5. IL-33 signaling controls the development of red pulp macrophages (RPM). A, Representative examples of flow cytometry staining and quantification of RPM and pre-RPM (% among CD11c^{low} Ly6G^{low} NK1.1^{low} SSC-A^{low} cells) in spleens of wild type (WT) and *Il33*$^{–/–}$ mice, 9 mice per group. Data are representative of at least 5 independent experiments. **B**, Representative examples and quantification (among CD11c^{low} Ly6G^{low} NK1.1^{low} SSC-A^{low} cells) of flow cytometry staining for splenic pre-RPM (CD11b^{+} F4/80^{lo}) and RPM (CD11b$^{lo/–}$ F4/80^{hi}) in mice injected intraperitoneally once a day for 3 days with either phosphate buffered saline (PBS), IL-33 (1 μg), hemin (500 μg), or IL-33 + hemin, 5 mice per group. Data are representative of at least 4 independent experiments. **C**, Representative examples and quantification of splenic RPM (CD11b$^{lo/–}$ EMR1^{hi}) in wild-type and *Il33*$^{–/–}$ rats. Each dot in **C** represents a separate rat ****P<0.001, data are representative of 2 independent experiments.

Figure S6

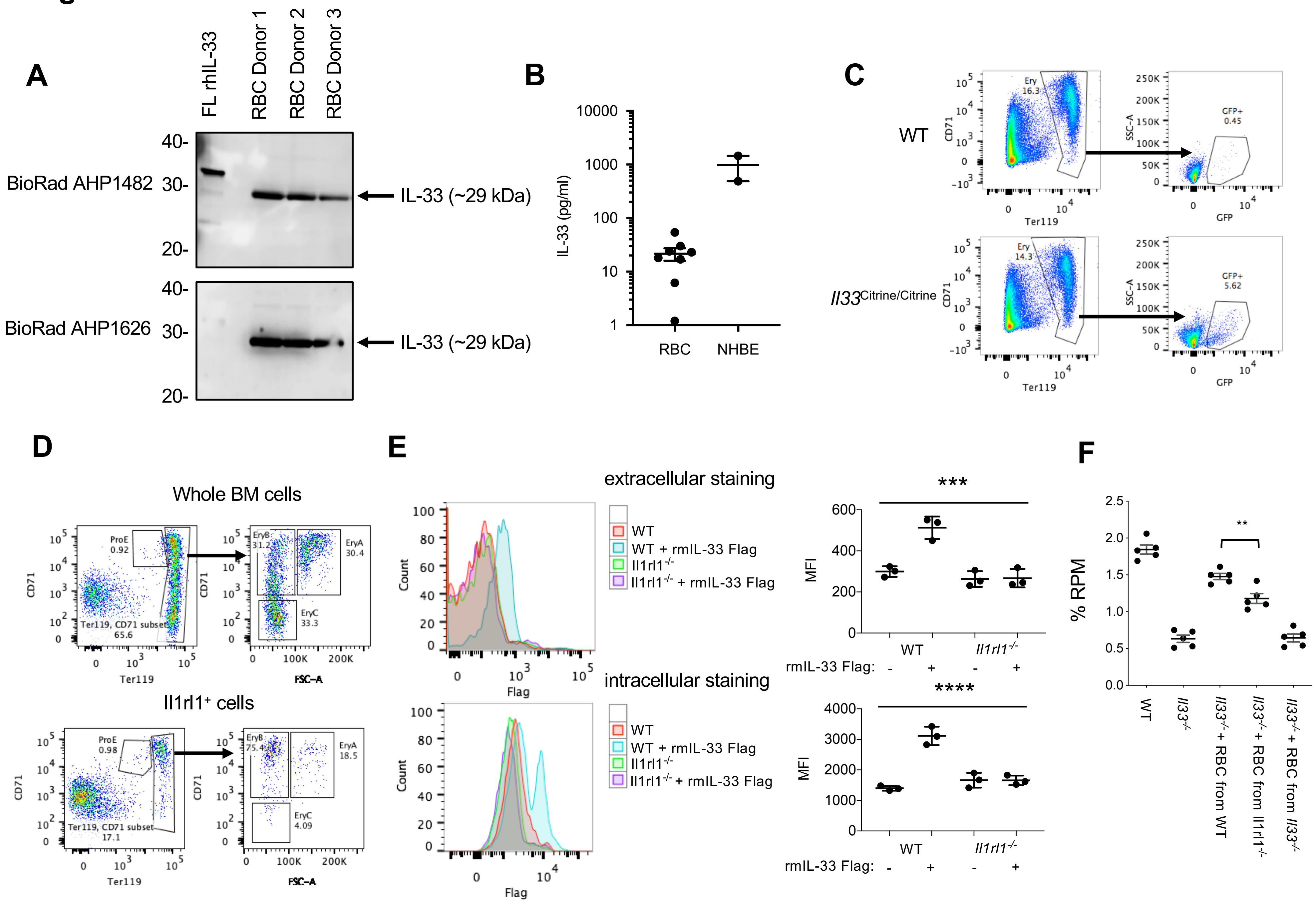

A
FL rhIL-33
RBC Donor 1
RBC Donor 2
RBC Donor 3
BioRad AHP1482
BioRad AHP1626
40-
30-
20-
IL-33 (~29 kDa)
B
IL-33 (pg/ml)
10000
1000
100
10
1
RBC
NHBE
C
WT
Il33Citrine/Citrine
CD71
Ter119
Ery
16.3
14.3
SSC-A
GFP
GFP+
0.45
5.62
D
Whole BM cells
Il1rl1+ cells
ProE
0.92
0.98
Ter119, CD71 subset
65.6
17.1
EryB
31.2
75.4
EryA
30.4
18.5
EryC
33.3
4.09
FSC-A
E
extracellular staining
intracellular staining
WT
WT + rmIL-33 Flag
Il1rl1-/-
Il1rl1-/- + rmIL-33 Flag
Count
Flag
MFI
***
****
rmIL-33 Flag:
F
% RPM
**
WT
Il33-/-
Il33-/- + RBC from WT
Il33-/- + RBC from Il1rl1-/-
Il33-/- + RBC from Il33-/-

Figure S6 related to Figure 5. IL-33 in red blood cell (RBC) progenitors and mature RBCs. **A**, Detection of IL-33 in human red blood cell (RBC) lysates using western blotting and 2 different anti-IL-33 antibodies. Recombinant full-length human IL-33 is included as a positive control. **B**, Quantification of IL-33 protein in RBC lysates. Normal human bronchial epithelial cells (NHBE) are used as positive controls. **C**, Representative examples of flow cytometry showing *Il33*-Citrine^{+} RBC progenitors (Ter119^{+}/CD71^{+} or CD71^{-}) in RBC-depleted bone marrow of wild type (WT) and *Il33*$^{Citrine/Citrine}$ reporter mice. **D**, Representative examples of flow cytometry staining for pro-erythroid cells (ProE, Ter119^{int}/CD71^{hi}), and erythroblast subsets A (less mature, Ter119^{hi} CD71^{hi} FSC^{hi}), B (intermediate maturation, Ter119^{hi} CD71^{hi} FSC^{lo}) and C (most mature, Ter119^{hi} CD71$^{lo/-}$ FSC^{lo}) in the bone marrow of wild type mice. IL1RL1 (ST2)$^{+}$ cells are enriched in pro-erythroid cells and erythroblast B subset. **E**, recombinant murine IL-33-Flag was incubated with whole bone marrow cells of WT and *Il1rl1*$^{-/-}$ mice for 60 min, which was followed by extracellular and intracellular staining for Flag. Results are gated on Ter119^{hi} cells (erythroblasts) and mean fluorescence intensity (MFI) is shown. Data are representative of 3 independent experiments. **F**, Quantification (% among CD11c^{low} Ly6G^{low} NK1.1^{low} SSC-A^{low} cells) of splenic RPM (CD11b$^{lo/-}$ F4/80^{hi}) by flow cytometry in WT and *Il33*$^{-/-}$ mice. Some *Il33*$^{-/-}$ mice were reconstituted with either WT, *Il33*$^{-/-}$, or *Il1rl1*$^{-/-}$ RBCs (see Methods) prior to assessment of splenic RPM. **P<0.05, ***P<0.01, ****P<0.001.

Figure S7

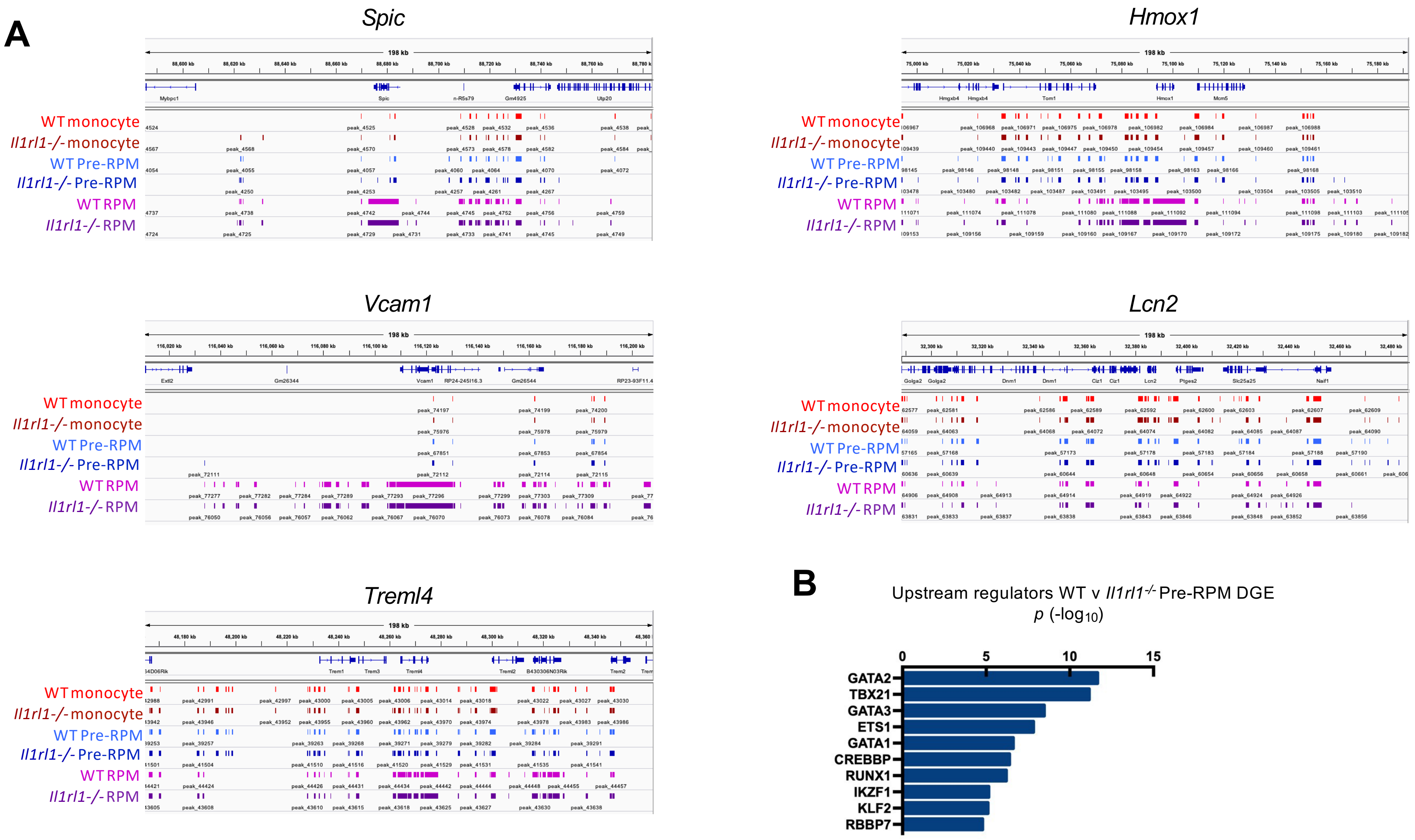

Figure S7 related to Figures 6 and 7. ATAC-sequencing and RNA-sequencing of splenic monocytes, pre-RPM and RPM. A, Examples of peak calling of accessible chromatin by ATAC-seq from splenic monocytes (CD11b^{hi} F4/80$^{–}$), pre-RPM (CD11b^{hi} F4/80^{lo}) and RPM (CD11b$^{lo/–}$ F4/80^{hi}) from WT and *Il1rl1*$^{–/–}$ mice, n=4 per group. **B,** Top upstream transcriptional regulators for differentially expressed genes (DGE) in WT and *Il1rl1*$^{–/–}$ pre-RPM (adjusted p-value <0.05), identified by Ingenuity pathway analysis.
